# Variation of Photosynthetic Induction in Major Horticultural Crops Is Mostly Driven by Differences in Stomatal Traits

**DOI:** 10.3389/fpls.2022.860229

**Published:** 2022-04-27

**Authors:** Ningyi Zhang, Sarah R. Berman, Dominique Joubert, Silvere Vialet-Chabrand, Leo F. M. Marcelis, Elias Kaiser

**Affiliations:** ^1^Horticulture and Product Physiology, Department of Plant Sciences, Wageningen University & Research, Wageningen, Netherlands; ^2^Biometris, Department of Mathematical and Statistical Methods, Wageningen University & Research, Wageningen, Netherlands

**Keywords:** induction, genotypic variation, light fluctuations, modeling, photosynthesis, Rubisco activation, stomatal opening

## Abstract

Under natural conditions, irradiance frequently fluctuates, causing net photosynthesis rate (*A*) to respond slowly and reducing the yields. We quantified the genotypic variation of photosynthetic induction in 19 genotypes among the following six horticultural crops: basil, chrysanthemum, cucumber, lettuce, tomato, and rose. Kinetics of photosynthetic induction and the stomatal opening were measured by exposing shade-adapted leaves (50 μmol m^–2^ s^–1^) to a high irradiance (1000 μmol m^–2^ s^–1^) until *A* reached a steady state. Rubisco activation rate was estimated by the kinetics of carboxylation capacity, which was quantified using dynamic *A* vs. [CO_2_] curves. Generally, variations in photosynthetic induction kinetics were larger between crops and smaller between cultivars of the same crop. Time until reaching 20–90% of full *A* induction varied by 40–60% across genotypes, and this was driven by a variation in the stomatal opening rather than Rubisco activation kinetics. Stomatal conductance kinetics were partly determined by differences in the stomatal size and density; species with densely packed, smaller stomata (e.g., cucumber) tended to open their stomata faster, adapting stomatal conductance more rapidly and efficiently than species with larger but fewer stomata (e.g., chrysanthemum). We conclude that manipulating stomatal traits may speed up photosynthetic induction and growth of horticultural crops under natural irradiance fluctuations.

## Introduction

Irradiance in canopies frequently fluctuates due to changes in solar angle, cloud movements, and wind-induced leaf movements (Pearcy, 1953; Kaiser et al., 2015). When irradiance increases, the rate of photosynthesis of a shade-adapted leaf does not immediately increase to a new steady-state level. Instead, leaf photosynthesis increases progressively, until it reaches a new steady-state level; this process is referred to as photosynthetic induction. The time needed for photosynthetic induction leads to a potential carbon loss, as during this time period, leaf photosynthesis operates below its steady-state rate (Mott and Woodrow, 2000; Salter et al., 2019; Acevedo-Siaca et al., 2020). Thus, speeding up photosynthetic induction may increase the yields of crops grown under fluctuating light (Slattery et al., 2018; Tanaka et al., 2019; Acevedo-Siaca et al., 2020).

Photosynthetic induction is generally considered to be limited by three main processes: (1) photoactivation of enzymes involved in the regeneration and production of ribulose 1,5-bisphosphate (RuBP), (2) increase in the activation state of Rubisco, and (3) stomatal opening (Pearcy, 1953; Kaiser et al., 2015, 2018). Mesophyll conductance may also limit photosynthetic induction (especially when transitioning from darkness to light), but the importance of mesophyll conductance limitation for photosynthetic induction is currently under debate (De Souza et al., 2020; Liu et al., 2021; Sakoda et al., 2021). Large genotypic variation in photosynthetic induction rates has previously been found in many crop species (McAusland et al., 2016; Salter et al., 2019; Acevedo-Siaca et al., 2020; Yamori et al., 2020). For example, in rice, soybean, and cassava, the integrated net photosynthesis rate (*A*) during the first 5 min after a switch from low to high irradiance was affected by genotypic variation (Soleh et al., 2017; Acevedo-Siaca et al., 2020; De Souza et al., 2020). Quantifying the genotypic variations of photosynthetic induction and identifying the relevant physiological traits can help with trait selection for breeding high-yielding cultivars with optimized photosynthetic induction.

The activation of enzymes involved in RuBP regeneration is thought to be complete within the first 1–2 min of photosynthetic induction (Pearcy, 1953); the extent of this limitation was found to be relatively similar among closely related wheat genotypes (Salter et al., 2019). The extent to which Rubisco activation and stomatal opening limit photosynthetic induction vary more strongly among species, and these two limitations are often interlinked. For example, in rice, wheat, and soybean, photosynthetic induction was found to be limited by the rate of Rubisco activation and presumably driven by concentrations of Rubisco and Rubisco activase (Soleh et al., 2016; Salter et al., 2019; Acevedo-Siaca et al., 2020). However, some studies also showed a strong role of stomatal conductance (*g*_*s*_) in the photosynthetic induction of rice and wheat (Adachi et al., 2019; McAusland et al., 2020; Qu et al., 2020; Yamori et al., 2020). In recent years, the activation of Rubisco during photosynthetic induction has been approximated by estimating the dynamics of maximum Rubisco carboxylation rate (*V*_*cmax*_) during photosynthetic induction through dynamic *A* vs. intercellular CO_2_ concentration (*C*_*i*_) curves (Soleh et al., 2016; Taylor and Long, 2017; Salter et al., 2019). This requires a measure of photosynthetic induction at several *C*_*i*_, allowing for the estimation of the time constants that describe several phases of *V*_*cmax*_ induction and the kinetics of changes in electron transport rate at high irradiance (Soleh et al., 2016; Taylor and Long, 2017; Salter et al., 2019).

In many species, such as cassava, tomato, Arabidopsis, and in some tropical trees and shrubs, photosynthetic induction tends to strongly correlate with stomatal traits (e.g., initial *g*_*s*_ in low irradiance or stomatal opening rate) (Valladares et al., 1997; Allen and Pearcy, 2000; Kaiser et al., 2016, 2020; De Souza et al., 2020). Increasing *g*_*s*_ has been found to speed up the photosynthetic induction in rice and tomato (Kaiser et al., 2020; Sakoda et al., 2020; Yamori et al., 2020). Stomatal anatomy (e.g., stomatal density and size) affects *g*_*s*_, including its kinetics. Smaller stomata tend to show lower initial *g*_*s*_ at low irradiance, but faster opening and closure kinetics (Drake et al., 2013; Giday et al., 2013; Kardiman and Ræbild, 2018; Zhang et al., 2019). However, this inverse stomatal size–speed relationship is not conserved across species, as guard cell shape (elliptical, dumbbell) and guard cell cytoskeleton, cell wall elasticity, number and activity of transporters, or ion channels, also affect the rapidity of the stomatal response (Elliott-Kingston et al., 2016; McAusland et al., 2016; Lawson and Vialet-Chabrand, 2019).

Studies investigating the photosynthetic induction have so far mostly been conducted on the major field crops (e.g., rice, wheat, and soybean) and species in forestry eco-systems (Valladares et al., 1997; Allen and Pearcy, 2000; McAusland et al., 2016; Salter et al., 2019; Acevedo-Siaca et al., 2020), which leave a knowledge gap for other economically important species, such as tomato, cucumber, lettuce, and chrysanthemum. Despite the fact that irradiance fluctuations mostly occur under open-field conditions, irradiance in greenhouses can also fluctuate substantially (Supplementary Figure 1; Marcelis et al., 2018). Irradiance fluctuations in greenhouses are caused by the movement of the sun and cloud, both of which affect the shade cast by the greenhouse structure, including shading screens and supplemental lighting and canopy self-shading. An important distinction between the open fields and greenhouses is a near-complete lack of wind in the latter, which presumably reduces the frequency of sunlight fluctuations, and increases their duration, in the greenhouse. Crop growth in greenhouses is often source-limited, i.e., limited by crop photosynthesis (Marcelis, 1994); hence, greenhouse crops that respond to irradiance fluctuations with high efficiency are likely to show increased growth rates. Despite this substantial relevance of dynamic photosynthesis for crop growth in greenhouses, studies on the genotypic variation of photosynthetic induction so far have not included the major greenhouse crops.

The objective of this study was to quantify the genotypic variation of photosynthetic induction in some of the world’s major horticultural crops, such as tomato, cucumber, rose, chrysanthemum, lettuce, and basil. Furthermore, we aimed to elucidate the influence of the main factors that affect the rapidity of photosynthetic induction: Rubisco activation and stomatal opening, including the role of stomatal anatomy.

## Materials and Methods

### Plant Material and Growth Conditions

The experiment was conducted from 28 January to 12 June 2020, in a compartment (8 × 8 m) of a Venlo-type glasshouse located in Wageningen, the Netherlands (52°N, 6°E). Four growth tables were situated in the compartment. All genotypes were grown in the same compartment to avoid artifacts caused by different growth conditions. In total, 19 genotypes of six horticultural crop species were used, including two flower crops, chrysanthemum (*Chrysanthemum morifolium*) and rose (*Rosa hybrida*); two fruit vegetables, cucumber (*Cucumis sativus L*.) and tomato (*Solanum lycopersicum L*.); and two leafy vegetables, basil (*Ocimum basilicum*) and lettuce (*Lactuca sativa L*.; Table 1). Cultivars for each crop were chosen based on their commercially relevant traits as horticultural merchandise: cultivars of the flower crops differed in flower color and number, those of fruit vegetables differed in fruit size, and those of leafy vegetables differed in leaf color and texture. For basil, cucumber, lettuce, and tomato, seeds were sown in rockwool plugs (diameter: 2 cm). Following germination, the seedlings were transferred to rockwool cubes (10 × 10 cm). Chrysanthemum plants were grown in plastic pots (diameter: 14 cm) filled with potting soil. Rose plants were grown in rockwool cubes (7 × 7 cm).

**TABLE 1:** Horticultural genotypes used in the experiment, with abbreviations used throughout the text in brackets, and starting plant materials.

Crop	Commercial cultivar name (abbreviation)	Starting material
Basil	Eleonora (BEL)^1^; Emily (BEM)^1^; Rosie (BR)^1^	Seeds
Chrysanthemum	Anastasia (CHA)^2^; Baltica (CHB)^2^; Radost (CHR)^2^; Yellow Zembla (CHY)^2^	Cuttings
Cucumber	Hipower (CUH)^3^; Mewa (CUM)^4^; Proloog (CUP)^4^	Seeds
Lettuce	Cecilia (LC)^4^; Gardia (LGA)^4^; Gilmore (LGI)^4^	Seeds
Rose	Apple Park (RAP)^5^; Avalanche (RAV)^6^; Red Naomi (RRN)^5^	Cuttings
Tomato	Brioso (TB)^4^; Merlice (TM)^7^; Sweeterno (TS)^4^	Seeds

*^1^Provided by Enza Zaden, NL; ^2^provided by Deliflor, NL; ^3^provided by Nunhems (Basf), NL; ^4^provided by Rijk Zwaan, NL; ^5^provided by Schreurs, NL; ^6^provided by Dümmen Orange, NL; ^7^provided by Bayer Crop Science, NL.*

For basil, cucumber, lettuce, and tomato, the seeds were sown weekly. For chrysanthemum and rose, the plants were cut back weekly at the third or fourth node, counting from the base, to allow for the formation of a new axillary bud. Two weeks after sowing seeds or cutting back plants, two plants per genotype were placed on a growth table in a grid of four rows (distance between rows: 50 cm; distance between plants within the row: 30 cm). Plant positions were randomized two times per week to minimize any effects of a heterogeneous climate in the greenhouse compartment on plant growth. Plants were placed on the growth table for 2–3 weeks (i.e., 4–5 weeks after sowing seeds or cutting back plants), after which the measurements were conducted. This protocol was repeated weekly until data of 7–9 replicates per genotype had been collected.

A mixture of high-pressure sodium lamps (600 W, Philips, Eindhoven, Netherlands) and white light-emitting diodes (LEDs) (GreenPower LED toplighting module, Signify, Eindhoven, Netherlands) were used between 02:00 and 18:00 (a photoperiod of 16 h). Lamps were switched on during the photoperiod whenever global radiation (GR) outside the greenhouse dropped below 150 W m^–2^ and were switched off when GR > 250 W m^–2^. Photosynthetically active radiation (PAR) from both lamp types combined was, on average, 226 ± 16 μmol m^–2^ s^–1^ at the canopy level (mean ± S.D.; Supplementary Figure 2). A shading screen (HARMONY 4215 O FR, Ludvig Svensson, Hellevoetsluis, Netherlands) was closed when GR > 600 W m^–2^ and was opened when GR < 500 W m^–2^. Day and night temperatures were set to 20 and 19°C, respectively. Relative humidity was set to 60%. Climate settings were designed to provide reasonably optimal growth conditions for all genotypes, in discussion with greenhouse cultivation experts at the Wageningen University. Average values of daily PAR (from both solar light and supplemental lamps), air temperature, relative humidity, and [CO_2_] inside the greenhouse during the experiment were 241 ± 48 μmol m^–2^ s^–1^, 21.5 ± 1.4°C, 63 ± 6%, and 445 ± 11 ppm, respectively (mean ± SD; Supplementary Figure 3). Plants were irrigated four times per day between 7:00 and 19:00 with a customized nutrient solution suitable for all six greenhouse crops (pH: 6.3; EC: 2.2 mS cm^–1^; Supplementary Table 1).

### Gas Exchange Measurements

Net photosynthesis rate (*A*) and stomatal conductance to water vapor (*g*_*s*_) were measured on the youngest fully expanded leaf, using a gas exchange system (LI-6800, Li-Cor Bioscience, Lincoln, NE, United States) equipped with a 6 cm^2^ leaf chamber fluorometer. No correction for the leaf area was needed for any of the gas exchange measurements as the leaves always fully filled the leaf chamber. All measurements were performed at an air temperature of 23°C, relative humidity of 65%, and a flow rate of air through the system of 500 μmol s^–1^. Irradiance was provided by a mixture of red (90%) and blue (10%) LEDs in the fluorometer. Before any gas exchange measurement, single plants were preconditioned to a low irradiance (ca. 50 μmol m^–2^ s^–1^) for 40–60 min in the greenhouse compartment, using a custom-built shading construction. The shading construction was covered by opaque plastic films and with LEDs installed at the top, which produced an irradiance at around 50 μmol m^–2^ s^–1^ with 90% red and 10% blue colors. Preadaptation under the shading construction for 40–60 min was applied to ensure that during subsequent gas exchange measurements, the leaves were sufficiently shade-adapted to produce comparable data.

Photosynthetic induction was measured under a range of [CO_2_]: 50, 100, 250, 400, 600, 800, and 1,000 ppm. At 400 ppm of CO_2_, the leaf was first exposed to a low irradiance of 50 μmol m^–2^ s^–1^ for 30 min in the gas exchange chamber, after which the irradiance was increased in a single step to a high level (1,000 μmol m^–2^ s^–1^) for an additional 30 min. A low irradiance rather than darkness was used for the initial light conditions, as in natural environments; shade-adapted leaves are often suddenly exposed to high light (due to cloud movements or wind), whereas the exposure of an entirely dark-adapted leaf to a high irradiance is unlikely in nature and greenhouses. Gas exchange data was logged every 2 s. If a steady-state *A* value was not reached after 30 min under high irradiance, measurements continued until *A* reached a steady state. For other [CO_2_], the leaf was clamped into the cuvette at 50 μmol m^–2^ s^–1^ for 5 min, after which the irradiance was increased to 1,000 μmol m^–2^ s^–1^ for 15 min. All measurements ([CO_2_] × genotype) were randomized. Every week, a group of new plants (one per genotype) was chosen and measurements at different [CO_2_] were randomized among these plants during the day, to avoid potential diurnal effects being entangled with treatment effects. Once photosynthetic induction was measured on a given plant, the particular plant was not used for another measurement for at least 40 min, to avoid interference from previous conditions. All gas exchange measurements were done between 8:00 and 16:00 h.

### Leaf Anatomical and Physiological Measurements

Samples to measure the stomatal size and density were taken on the same leaf used for gas exchange measurements. In addition, leaf light absorptance and chlorophyll and carotenoid contents were measured.

#### Stomatal Imprints

Stomatal imprints were taken after the final gas exchange measurement on a given plant had been completed. Imprints were taken using a silicone impression material (Zhermack, Badia Polesine, Italy), with two technical replicates on the abaxial side and two technical replicates on the adaxial side of each leaf. The silicon was allowed to fully dry on the leaf before it was removed gently. Clear nail polish was applied to the imprint and allowed to dry. The dry nail polish was viewed under a microscope (Leitz Aristoplan; Leica Microsystems, Wetzlar, Germany) and photographed at 25X and 40X magnification (Digital-Sight DS-Ri-1; Nikon, Tokyo, Japan). Images were analyzed with ImageJ, using the CellCounter and ObjectJ plugins (National Institute of Health, Bethesda, MD, United States).

#### Leaf Optical Properties

Leaf reflectance and transmittance were measured in the range of 400–700 nm for both adaxial and abaxial sides of the leaf. The measurement system consisted of two integrating spheres, each connected to a spectrometer and a custom-made light source (Hogewoning et al., 2010).

#### Leaf Chlorophyll and Carotenoid Contents

After the completion of gas exchange measurements, a leaf sample of 0.75 cm^2^ was taken from each leaf and stored at –80°C. Samples were extracted with 1.5 ml of *N,N*-Dimethylformamide (DMF) at –20°C for approximately 2 weeks (Wellburn, 1994). The absorption of the DMF solution was measured at 480, 647, 664, and 750 nm for chlorophyll *a*, chlorophyll *b*, and carotenoid contents, using a SpectraMax iD3 Microplate Reader (software version 1.2.0.0, Molecular Devices, San Jose, CA, United States) or a Genesys 15 UV-Visible spectrophotometer (Thermo Fisher Scientific, Waltham, United States). Concentrations were calculated according to the study by Wellburn (1994).

### Calculations

#### Rate of *A* Induction

The induction state of photosynthesis (IS) was calculated as follows:


(1) $$IS\left(t\right)=\frac{A\left(t\right)-A_{i}}{A_{f}-A_{i}}$$

where *A(t)* (μmol CO_2_ m^–2^ s^–1^) is CO_2_ assimilation rate at time *t* and 400 ppm CO_2_; *A*_*i*_ (μmol CO_2_ m^–2^ s^–1^) is initial *A* at low irradiance (average *A* measured in the last minute of low irradiance at 400 ppm CO_2_); *A*_*f*_ (μmol CO_2_ m^–2^ s^–1^) is final steady-state *A* reached in high irradiance, at 400 ppm CO_2_. The times to reach 20% (T_20_), 50% (T_50_), and 90% (T_90_) of full induction state were determined as the moments at which IS was the closest to these percentages, based on the IS time course generated from Eq. 1.

#### Rate of *V*_*cmax*_ and *J* Induction

Based on photosynthetic induction measurements at different [CO_2_], *A*/*C*_*i*_ curves were generated from the data obtained every 2 s under different [CO_2_]. First, respiration rate (*R*_*d*_) was estimated according to the study by Laisk (1977), i.e., *R*_*d*_ was identified as the intercept with the *y*-axis of the common intersection point of *A* vs. *C*_*i*_ at low and high irradiance, using the last data points measured under low and high irradiance at atmospheric [CO_2_] below 400 ppm. Then, the model of Farquhar et al. (1980) (the FvCB model) was fitted to each *A*/*C*_*i*_ curve to provide transient values of *V*_cmax_ and electron transport rate (*J*) during photosynthetic induction (Supplementary Method 1). The response of *V*_cmax_ induction during the first 15 min after exposure to high irradiance was fitted to an empirical model that represents a two-phase exponential function of time (Salter et al., 2019):


(2) $$\begin{array}{c} V_{cmax}\left(t\right)=V_{mi}+\left(V_{mf}-V_{mi}\right)\{f\left[1-exp\left(-\frac{t}{\tau_{fast}}\right)\right]\\ \;+\left(1-f\right)\left[1-exp\left(-t/\tau_{slow}\right)\right]\} \end{array}$$

where *V*_cmax_*(t)* is *V*_cmax_ at time *t*; *V*_mi_ is initial *V*_cmax_ after exposure to high irradiance; *V*_*mf*_ is final *V*_cmax_ after 15 min of high irradiance exposure; τ_fast_ and τ_slow_ are time constants for the fast and slow phase of *V*_cmax_ induction; *f* is a weighting factor (value: 0–1).

#### Transient Stomatal and Non-stomatal Limitations

Transient stomatal and non-stomatal limitations during photosynthetic induction were calculated based on an elimination approach. First, using the FvCB model, instantaneous *A* during photosynthetic induction was calculated every 2 s, with estimated *V*_cmax_ and *J* and measured *g*_s_ every 2 s (i.e., *V*_*mt*_, *J*_*t*,_ and *g*_s,t_) as input parameters. Calculated *A* was compared with the measured *A* during photosynthetic induction to ensure that model outputs accurately predicted the observed data before applying the elimination approach (Supplementary Presentation 1). In case of mismatches, values of *V*_mt_ and *J*_t_ were optimized to improve model predictions. Photosynthesis rate as affected by transient stomatal limitation (*A*_s_) was calculated by removing the transient limitations of Rubisco and electron transport rate changes, by using final *V*_cmax_ and *J* (*V*_*mf*_ and *J*_f_) at high irradiance and instantaneous *g*_s_ (*g*_s,t_) during induction (Eq. 3; Wang and Jarvis, 1993).


(3) $$A_{s}=\text{min}\left\{A_{c}\left(V_{mf},g_{s,t}\right),A_{j}\left(J_{f},g_{s,t}\right)\right\}$$

In this case, any difference between *A*_s_ and *A*_f_ can be seen as caused by incomplete stomatal opening during induction. Stomatal limitation (*L*_s_) was then calculated using Eq. 4:


(4) $$L_{s}=\frac{A_{f}-A_{s}}{A_{f}-A_{i}}\cdot 100$$

Photosynthesis rate as affected by transient non-stomatal limitation (*A*_ns_) was calculated by using instantaneous *V*_cmax_ and *J* (*V*_mt_ and *J*_t_) during induction and final *g*_s_ (*g*_s,f_) reached at high irradiance (Eq. 5; Wang and Jarvis, 1993).


(5) $$A_{ns}=\text{min}\left\{A_{c}\left(V_{mt},g_{s,f}\right),A_{j}\left(J_{t},g_{s,f}\right)\right\}$$

In this case, any difference between *A*_ns_ and *A*_f_ can be seen as caused by the incomplete induction of *V*_cmax_ and *J*. Nonstomatal limitation (*L*_ns_) was then quantified using Eq. 6.


(6) $$L_{ns}=\frac{A_{f}-A_{ns}}{A_{f}-A_{i}}\cdot 100$$

#### Kinetics of *g*_*s*_ Responses

The response of *g*_s_ to a single step change in light intensity was quantified using a dynamic *g*_s_ model (Vialet-Chabrand et al., 2013; McAusland et al., 2016). The model describes the temporal response of *g*_s_, using a time constant (*k*, min), an initial time lag (λ, min), and a steady-state *g*_s_ (*g*_s,f_, mol m^–2^ s^–1^) reached a given irradiance:


(7) $$g_{s}=\left(g_{s,f}-g_{s,i}\right)e^{-e^{\left(\frac{\lambda -t}{k}+1\right)}}+g_{s,i}$$

where *g*_s,i_ is the initial *g*_s_ value at low irradiance (average *g*_s_ measured in the last minute of low irradiance at 400 ppm CO_2_). The time constant, *k*, describes the rapidity of the *g*_s_ response, independent of the amplitude of variation in *g*_s_. The value, *e* is Euler’s number (2.71828). Based on *k* and *g*_s,f_, the maximum slope of the *g*_s_ response to a step-change in irradiance (*Sl*_*max*_, μmol m^–2^ s^–2^), which combines the rapidity and amplitude of the response, was calculated:


(8) $$Sl_{max}=\frac{g_{s,f}-g_{s,i}}{k\cdot e}$$

#### Theoretical Maximum Stomatal Conductance

The maximum stomatal conductance to water vapor (*g*_s,max_) when all stomates open to their maximum extent was calculated based on the studies by Franks and Farquhar (2001) and Franks and Beerling (2009):


(9) $$g_{s,max}=\frac{d\cdot SD\cdot a_{max}}{v\left(l+\frac{\pi }{2}\sqrt{\frac{a_{max}}{\pi }}\right)}$$

where *d* is the diffusivity of water vapor in the air (24.9 × 10^–6^ m^2^ s^–1^); *v* is the molar volume of air (24.4 × 10^–3^ m^3^ mol^–1^); SD is stomatal density; *a*_max_ is the maximum pore area and is approximated as π(ρ/2)^2^, where ρ is stomatal pore length and *l* is stomatal pore depth (assumed to be equal to guard cell width). Both ρ and guard cell width were measured from stomatal imprints (Supplementary Table 2). Based on the stomatal density and length obtained from abaxial and adaxial leaf surfaces, *g*_s,max_ per leaf surface was calculated. Then, *g*_s,max_ for a specific genotype was calculated as the sum of *g*_s,max_ for both leaf surfaces.

#### Kinetics of Stomatal Pore Area Increase

When substituting *g*_*s,max*_ in Eq. 9 with *g*_s_ obtained from gas exchange measurements, *a*_*max*_ represents the average stomatal pore area *a* across the leaf surface. Thus, the stomatal pore area and its kinetics during the stomatal opening were quantified by solving *a* from Eq. 9 (refer to details in Supplementary Method 2):


(10) $$a={\left(\frac{\frac{\sqrt{\pi }}{2}\cdot g_{s}\cdot v+\sqrt{\frac{\pi }{4}{\left(g_{s}\cdot v\right)}^{2}+4*SD*gs*v*l}}{2\cdot SD\cdot d}\right)}^{2}$$

It is important to note that the relationship between *a* and *g*_*s*_ is not linear, which can result in differences in temporal kinetics between both traits.

#### Coefficient of Variation

To evaluate the variation of traits among genotypes, the coefficient of variation (CV, %) was calculated:


(11) $$CV=\frac{X_{sd}}{X_{avg}}\cdot 100$$

where *X*_sd_ and *X*_avg_ are, respectively, the standard deviation and mean value of the genotype-specific average of a given trait across all 19 genotypes.

### Statistical Analysis

Using a nonlinear regression with the GAUSS method in PROC NLIN of SAS (SAS Institute Inc., Cary, NC, United States), parameters of the dynamic *g*_s_ model and *V*_cmax_ kinetics during *A* induction were estimated. Statistical analyses were conducted using R^1^. First, normality was tested using the Shapiro–Wilk test, and homogeneity was tested using Levene’s test to determine whether residuals showed equal variances. For traits that did not show equal variance, log transformation of data was applied. Differences between genotypes were detected using one-way ANOVA (*p* < 0.05), by taking into account, the different weeks of sowing/cutting as a block effect. When a significant difference was detected, a *post hoc* test was conducted for pairwise comparisons between genotypes, using Fisher’s Protected Least Significant Difference (LSD) test (*p* < 0.05).

## Results

### Genotypic Variation of *A* and *g*_s_ Responses to a Single-Step Change in Irradiance

**FIGURE 1:**
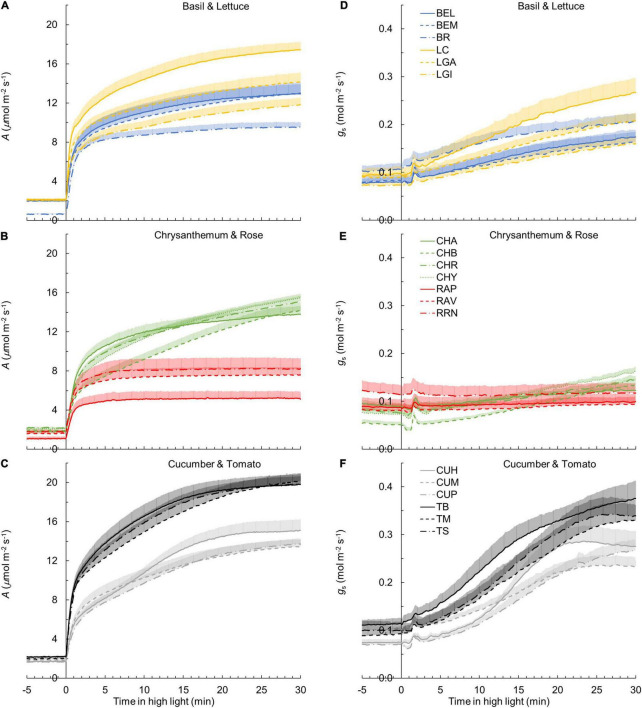
Leaf photosynthesis rate (*A*) **(A–C)** and stomatal conductance *g*_*s*_ **(D–F)** responses to a single-step change in irradiance in 19 horticultural genotypes of six species (**A,D**: basil and lettuce; **B,E**: chrysanthemum and rose; **C,F**: cucumber and tomato; refer to Table 1 for full names of genotypes). Time zero indicates the moment when irradiance was increased from 50 to 1,000 μmol m^–2^ s^–1^. *A* and *g*_*s*_ values were logged every 2 s. Line colors represent crop species, while line types differentiate between cultivars. Each curve represents the mean of 7–9 individual plants (mean + SE).

**FIGURE 2:**
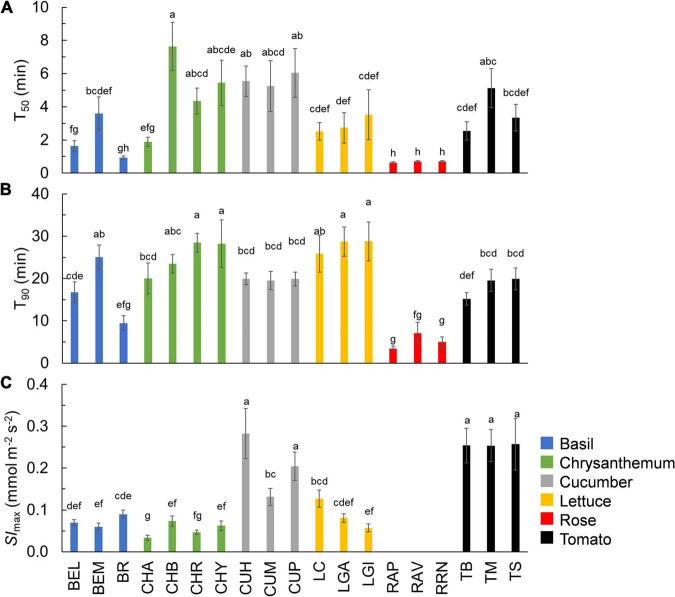
Time needed for *A* to reach 50% (**A:** *T*_50_) and 90% (**B:** *T*_90_) of full photosynthetic induction, as well as the maximum rate of stomatal opening after an increase in irradiance (**C:** *Sl*_max_) in 19 horticultural genotypes. Colors indicate crop species. Bars show means ± SE (*n* = 7–9). Letters indicate significant differences (*p* < 0.05). For *T*_50_, the result of the statistical test was based on log transformation of original data. *Sl*_max_ was not estimated for rose. Refer to Table 1 for full genotype names.

The kinetics of *A* induction varied substantially among the 19 horticultural genotypes tested (Figures 1A–C). Variation in the key traits of photosynthesis dynamics tended to be larger between different crop species than between cultivars of the same species (Figure 2). CV of T_20_, T_50_, and T_90_ was 55, 61, and 42%, respectively, while CV of average *A* during the first 300 s of induction (*A*_avg,300_) was 22% (Table 2). The rate of *A* induction in rose was the fastest as demonstrated by small values for T_50_ and T_90_ in all three rose cultivars (Figures 1B, 2A,B). Chrysanthemum and lettuce, on the other hand, tended to have the highest *T*_90_ values (Figure 2B) and thus showed relatively slow induction. Chrysanthemum also had high *T*_50_ values, except for cv. Anastasia (CHA) had a very low *T*_50_, whereas lettuce showed a relatively smaller *T*_50_ (Figure 2A). Tomato and cucumber had intermediate *T*_50_ and *T*_90_ (Figures 2A,B). Most crops showed a relatively small variation between cultivars, except for basil, which showed relatively large variations in *T*_50_ and *T*_90_ for its three cultivars (Figures 2A,B).

**TABLE 2:** Definition, unit, maximum, minimum, mean, and coefficient of variation (CV) for dynamic, steady-state, anatomical and physiological traits across 19 horticultural genotypes.

Trait	Definition	Unit	Max. (genotype)	Min. (genotype)	Mean	CV (%)
***Dynamic traits***						
*T*_20_	Time to reach 20% of full *A* induction	min	1.2 (CHB)	0.2 (RAP)	0.5	55
*T*_50_	Time to reach 50% of full *A* induction	min	7.6 (CHB)	0.6 (RAP)	3.4	61
*T*_90_	Time to reach 90% of full *A* induction	min	28.8 (LGI)	3.4 (RAP)	19.2	42
*A*_*avg,300*_	Average *A* during the first 300 s of induction	μmol m^–2^ s^–1^	10.9 (TB)	4.7 (RAP)	7.7	22
*g*_*s,avg,300*_	Average *g*_s_ during the first 300 s of induction	mol m^–2^ s^–1^	0.143 (TS)	0.052 (CHB)	0.099	22
iWUE_*avg,300*_	Average intrinsic water-use efficiency during the first 300 s of induction (*A*_*avg,300*_/*g*_*s,avg,300*_)	μmol CO_2_ (mol H_2_O)^–1^	117 (CHB)	42 (RAP)	84	21
*k*	Time constant for *g*_s_ response to irradiance change^1^	min	16.2 (LGI)	7.6 (CUH)	10.8	23
*Sl*_*max*_	Maximum rate of *g*_s_ response to irradiance change^1^	mmol m^–2^ s^–1^	0.28 (CUH)	0.03 (CHA)	0.13	68
λ	Initial time lag of *g*_s_ response to irradiance change^1^	min	7.4 (CUP)	0.1 (BR)	3.9	62
*f*	Weighting factor (between 0–1) for the fast and slow phase of *V*_cmax_ induction	–	0.7 (LGA)	0.4 (CHY)	0.5	18
τ_*fast*_	Time constant for fast phase of maximum Rubisco carboxylation rate (*V*_cmax_) induction	min	1.1 (CHA)	0.5 (LC)	0.7	22
τ_*slow*_	Time constant for slow phase of *V*_cmax_ induction	min	6.5 (TM)	3.1 (RAV)	4.8	22
***Steady-state traits***						
*A*_*i*_	Steady-state *A* at low irradiance	μmol m^–2^ s^–1^	2.2 (TS)	0.7 (BR)	1.9	21
*A*_*f*_	Steady-state *A* at high irradiance	μmol m^–2^ s^–1^	20.8 (TM)	5.7 (RAP)	14.4	30
Δ*A*	Difference between *A*_*f*_ and *A*_*i*_	μmol m^–2^ s^–1^	18.8 (TM)	4.5 (RAP)	12.5	33
*V*_*mi*_	*V*_cmax_ at the start of photosynthetic induction	μmol m^–2^ s^–1^	8.6 (CUP)	4.9 (BR)	7.0	16
*V*_*mf*_	*V*_cmax_ 15 min after start of photosynthetic induction	μmol m^–2^ s^–1^	65.9 (TB)	20.6 (RAP)	49.9	29
*g*_*s,i*_	Steady-state *g*_s_ at low irradiance	mol m^–2^ s^–1^	0.12 (RRN)	0.05 (CHB)	0.09	19
*g*_*s,f*_	Steady-state *g*_s_ at high irradiance	mol m^–2^ s^–1^	0.51 (TS)	0.10 (RAV)	0.25	46
***Leaf anatomical traits and pigments***						
SD_*ab*_	Stomatal density at abaxial leaf side	mm^–2^	340 (CUP)	40 (LGA)	124	78
SD_*ad*_	Stomatal density at adaxial leaf side	mm^–2^	267 (CUH)	0 (RAP, RAV, RRN)^2^	67	133
SS_*ab*_	Stomatal size at abaxial leaf side	μm^2^	1411 (CHB)	210 (CUP)	681	57
SS_*ad*_	Stomatal size at adaxial leaf side	μm^2^	1325 (CHR)	0 (RAP, RAV, RRN)^2^	540	81
*g*_*s,max*_	Theoretical maximum *g*_s_, if all stomates were to open to their maximum extent	mol m^–2^ s^–1^	5.0 (CUP)	1.3 (LGI)	2.5	50
Leaf_*chl*_	Leaf chlorophyll content^3^	mg m^–2^	222.0 (TM)	78.3 (LGA)	151.6	29
Chl *a*:*b*	Ratio of chlorophyll *a* to chlorophyll *b*	–	3.1 (LGA)	2.3 (BR)	2.7	7
Leaf_*caro*_	Leaf carotenoid content	mg m^–2^	28.4 (TM)	11.8 (BR)	19.1	25
Leaf_*abs*_	Leaf light absorptance^4^	–	0.89 (BR)	0.73 (LGA)	0.82	5

*Maximum and minimum values are average values of 6–9 replicates.*

*^1^Rose was excluded from estimations of k, Sl_max_, and λ, due to a lack of change between g_s,i_ and g_s,f_.*

*^2^Rose did not display stomata on the adaxial leaf side.*

*^3^Sum of chlorophyll a and chlorophyll b.*

*^4^Average value of both leaf surfaces.*

The kinetics of the *g*_s_ response to increases of irradiance also varied substantially among genotypes (Figures 1D–F). The value of *g*_*s*_ in rose barely responded to an irradiance increase; hence, *g*_s,i_ and *g*_*s,f*_ of rose cultivars were nearly identical (Figure 1E and Supplementary Figure 4). Therefore, the parameters representing the temporal response of *g*_s_ (*k*, λ, and *Sl*_max_) were not estimated for rose. Values for the CV of *k*, *Sl*_max_, and λ (among the remaining 16 genotypes) were, respectively, 23, 68, and 62% (Table 2). Both tomato and cucumber tended to have fast *g*_s_ increases, as well as exhibit stomatal oscillations (Figure 1F). Lettuce had medium *Sl*_max_, followed by chrysanthemum and basil, which had a relatively smaller *Sl*_max_ (Figure 2C). The CV of average *g*_*s*_ and water use efficiency in the first 5 min of *A* induction (*g*_s,avg,300_ and iWUE_avg,300_) were 22 and 21%, respectively, which were smaller than CV for most dynamic *g*_s_ parameters (λ and *Sl*_*max*_; Table 2).

Additionally, steady-state *A* and *g*_s_ varied strongly among genotypes (Figures 1A–C). *A*_i_ and *g*_s,i_ had a CV of ∼20% each (Table 2). For *A*_i_, basil had the lowest value and tomato the highest, while for *g*_s,i_, chrysanthemum showed the lowest value and rose showed the highest (Table 2). Steady-state *A* and *g*_s_ at high irradiance (*A*_f_ and *g*_f_) showed CV of 30 and 46%, respectively, with tomato showing the highest and rose the lowest *A*_f_ and *g*_s,f_ (Table 2).

### Kinetics of Biochemical Parameters and Transient Limitations During Photosynthetic Induction

**FIGURE 3:**
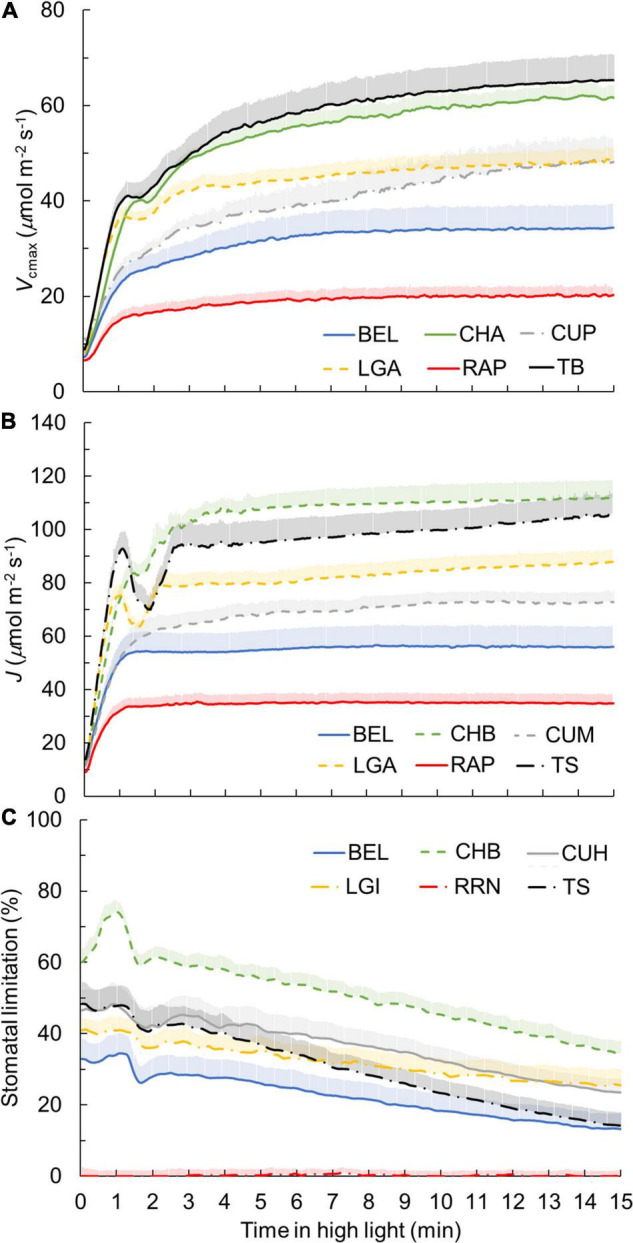
Dynamics of maximum Rubisco carboxylation rate *V*_cmax_ **(A)**, electron transport rate *J* **(B)**, and stomatal limitations for photosynthesis **(C)** during photosynthetic induction for six representative genotypes. Each curve represents the mean of 6–7 individual plants (mean + SE). Genotypes showing the smallest and largest value for a given trait among all genotypes, as well as cultivars with an intermediate response for their crop, are shown. Refer to Supplementary Figure 5 for representation of all 19 genotypes, as well as non-stomatal limitations for photosynthesis during photosynthetic induction.

**FIGURE 4:**
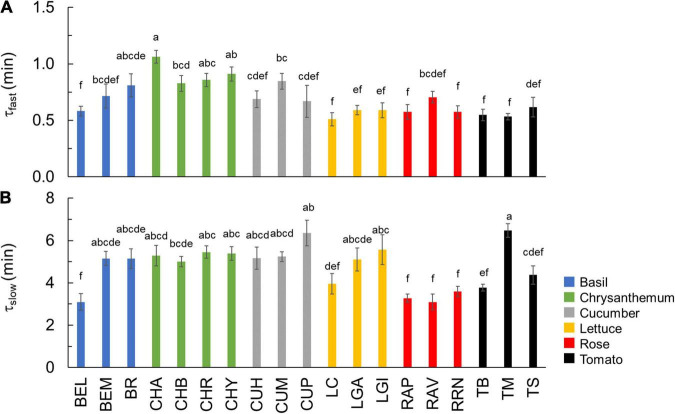
Time constants describing the fast (**A:** τ_*fast*_) and slow (**B:** τ_*slow*_) phase of *V*_cmax_ kinetics during photosynthetic induction. Colors indicate crop species. Bars show means ± SE (*n* = 6–7). Letters indicate significant differences (*p* < 0.05). Refer to Table 1 for full genotype names.

Based on dynamic *A* vs. *C*_i_ curves, the kinetics of *V*_cmax_ and *J*, as well as the stomatal and nonstomatal limitations to photosynthesis during *A* induction, were quantified (Figure 3 and Supplementary Figure 5). Both *V*_cmax_ and *J* induction kinetics varied between crops and cultivars of the same crop (Figures 3A,B and Supplementary Figures 5A,B). After 15 min in high irradiance, *V*_cmax_ and *J* of rose were the smallest, while tomato and chrysanthemum showed higher values for final *V*_cmax_ and *J* (Figures 3A,B). Interestingly, tomato and lettuce showed transient drops in *V*_cmax_ and *J* induction during the first 3 min after exposure to high irradiance (Figures 3A,B). Variations in time constants for *V*_cmax_ induction were smaller than those describing *A* induction. Both τ_fast_ and τ_slow_ had CV values of 22% (Table 2). Generally, τ_fast_ varied between 0.5 and 1 min, with chrysanthemum showing the largest τ_fast_ (Figure 4A). The values of τ_slow_ of basil, chrysanthemum, cucumber, and lettuce were generally around 5 min, and rose had the smallest τ_slow_ (3.1–3.6 min; Figure 4B). Surprisingly, large variations of τ_slow_ between cultivars were found in basil and tomato; basil cv. Eleonora (BEL) showed significantly smaller τ_slow_ than the other two basil cultivars, while tomato cv. Merlice (TM) showed a significantly larger τ_slow_ than the other two tomato cultivars (Figure 4B).

Within the first 15 min of exposure to high irradiance, transient non-stomatal and stomatal limitations of *A* induction showed substantial genotypic variation, with a greater variation in the level of transient stomatal limitation than in non-stomatal limitation (Figure 3C and Supplementary Figures 5C,D). Chrysanthemum showed the largest transient stomatal limitation among all crops, which went up to 70% during the first 1–2 min of induction and remained high (up to 40%) after 15 min in high irradiance (Figure 3C). Rose hardly exhibited any transient stomatal limitation during photosynthetic induction (Figure 3C), which can be explained by its non-responsive *g*_s_ to an irradiance increase (Figure 1E). Tomato showed a fast decrease in transient stomatal limitation (from ∼50% to ∼10% in 15 min) after an irradiance increase (Figure 3C). Transient non-stomatal limitation decreased sharply in the first 4–5 min after an irradiance increase (Supplementary Figure 5C). Most genotypes showed a transient non-stomatal limitation at around 10% after 15 min in high irradiance, except for cucumber, which still had ∼20% nonstomatal limitation after 15 min of high irradiance (Supplementary Figure 5C). Some crops (basil, chrysanthemum, and tomato) also showed relatively large variations between cultivars for transient stomatal and nonstomatal limitations (Supplementary Figures 5C,D).

### Genotypic Variation of Leaf Structural Traits

**FIGURE 5:**
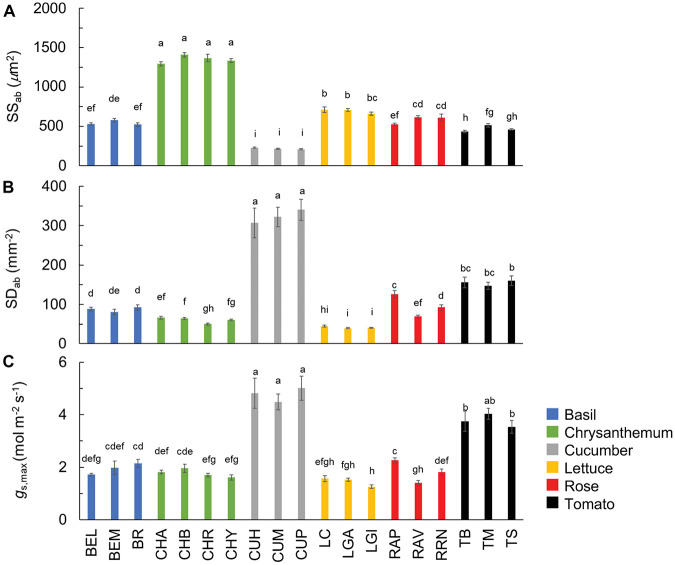
Stomatal size (**A**; SS_*ab*_) and density (**B**; SD_*ab*_) at the abaxial leaf side, and theoretical maximum stomatal conductance (**C**; *g*_*s,max*_) of all 19 horticultural genotypes. Colors indicate crop species. Bars show means ± SE (*n* = 7–9). Letters indicate significant differences (*p* < 0.05). Statistical test results of SS_*ab*_, SD_*ab*_, and *g*_*s,max*_ were based on log-transformation of the data. Refer to Table 1 for full genotype names.

Stomatal density and size showed large CV, especially adaxially, and this was partly due to the fact that the rose had no stomata at the adaxial side (Table 2). The CV of stomatal density and size at the leaf abaxial side were, respectively, 78 and 57% (Table 2). Generally, large variation in the stomatal density and size occurred between crop species, while the variation between cultivars was relatively small (Figures 5A,B). Chrysanthemum had the largest, and cucumber had the smallest stomata (Figure 5A). Both chrysanthemum and lettuce had low stomatal density, while cucumber had the highest stomatal density (Figure 5B). These large variations in the stomatal density and size resulted in large variation in theoretical maximum stomatal conductance (*g*_s,max_): the CV of *g*_s,max_ was 50%, with cucumber showing the highest *g*_s,max_ (up to ∼5 mol m^–2^ s^–1^), followed by tomato (up to ∼4 mol m^–2^ s^–1^), and lettuce, having the lowest *g*_s,max_ (∼1 mol m^–2^ s^–1^; Figure 5C and Table 2).

**FIGURE 6:**
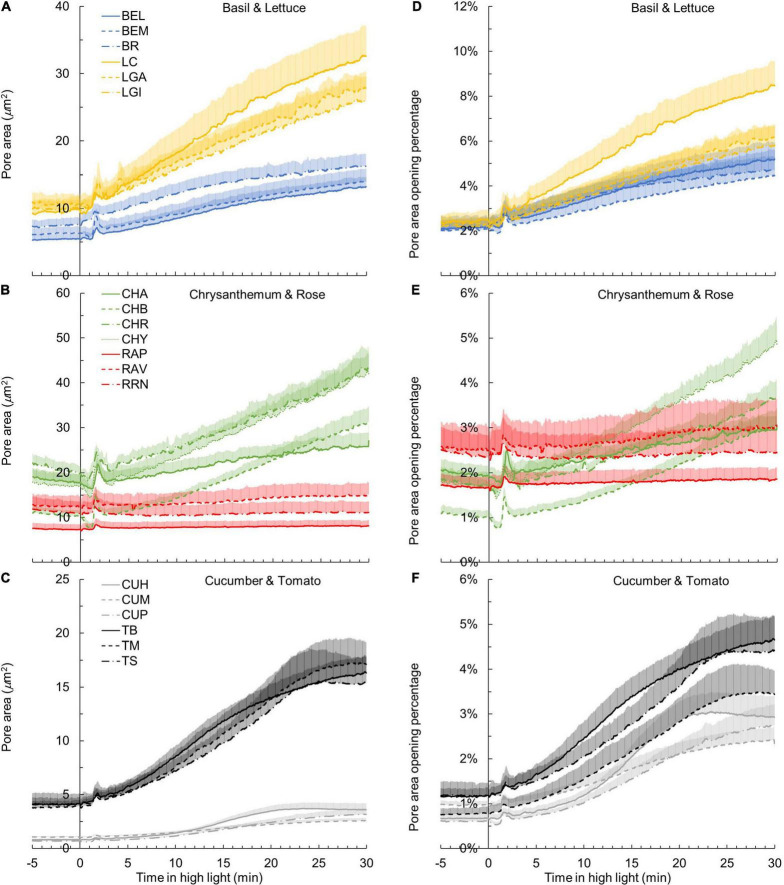
Kinetics of pore area opening **(A–C)** and pore area opening as a percentage of the theoretical maximum pore area **(D–F)** in response to a single-step change in irradiance in 19 horticultural genotypes (**A,D**: basil and lettuce; **B,E**: chrysanthemum and rose; **C,F**: cucumber and tomato; refer to Table 1 for full genotype names). Time zero indicates the moment when irradiance was increased from 50 to 1000 μmol m^–2^ s^–1^. Line colors represent crop species, while line types differentiate between cultivars. Each curve represents the mean of 7–9 individual plants (mean + SE).

Using values of *g*_*s,max*_ and observed *g*_s_ during photosynthetic induction (Figures 1D–F), absolute pore area opening was calculated. Kinetics of absolute pore area opening during *A* induction varied substantially between crops (Figures 6A–C). In cucumber leaves, individual pore area was found to be increased from ∼1 to ∼3 μm^2^ after 30 min in high irradiance, resulting in an increase of *g*_s_ from about 0.1 to 0.3 mol m^–2^ s^–1^ (Figure 6C and Supplementary Figure 4). The pore area of tomato was calculated to increase more strongly, from ∼4 to ∼16 μm^2^, leading to a *g*_s_ increase from about 0.1 to 0.4 mol m^–2^ s^–1^ (Figure 6C and Supplementary Figure 4). In contrast, the pore area of chrysanthemum and lettuce required a larger extent of opening to achieve a comparable *g*_s_ increase with cucumber and tomato from ∼18 to ∼36 μm^2^ in chrysanthemum and from ∼10 μm^2^ to ∼29 μm^2^ in lettuce (Figures 6A–C).

Surprisingly, when calculating the percentage of pore area opening relative to the maximum pore area, variation between crops was much smaller than for other traits (Figures 6D–F). During photosynthetic induction, all genotypes opened their stomata by less than 10% of the theoretical maximum pore area (calculated from pore length; Figures 6D–F). For example, the absolute pore area after 30 min in high irradiance reached ∼40 μm^2^ in chrysanthemum (which was the largest among all crops), which only accounted for 3–5% of the maximum pore area (Figures 6B,E). The pore area of cucumber only reached ∼3 μm^2^ after 30 min in high irradiance, which was also ∼3% of the maximum pore area (Figures 6C,F).

Leaf pigment concentrations showed relatively less variation among genotypes compared with most stomatal traits, with a CV of 25–30% for chlorophyll and carotenoid contents (Table 2). Since leaf color differed between cultivars (e.g., purple leaves in BR and brownish leaves in LGI), pigment types varied between crop species and cultivars (Supplementary Figures 6A,B). The chlorophyll *a*:*b* ratio showed little genotypic variation (CV: 7%), and an average of 2.7 across genotypes (Table 2). Leaf light absorptance was even more conserved, with a CV of 5%, the lowest value among all traits (Table 2). Small but significant differences in leaf light absorptance occurred between crop species, whereas variations between cultivars were not found, except for basil and lettuce which had cultivars (BR and LGI) with different leaf colors (Supplementary Figure 6C).

### Trait Correlations

**FIGURE 7:**
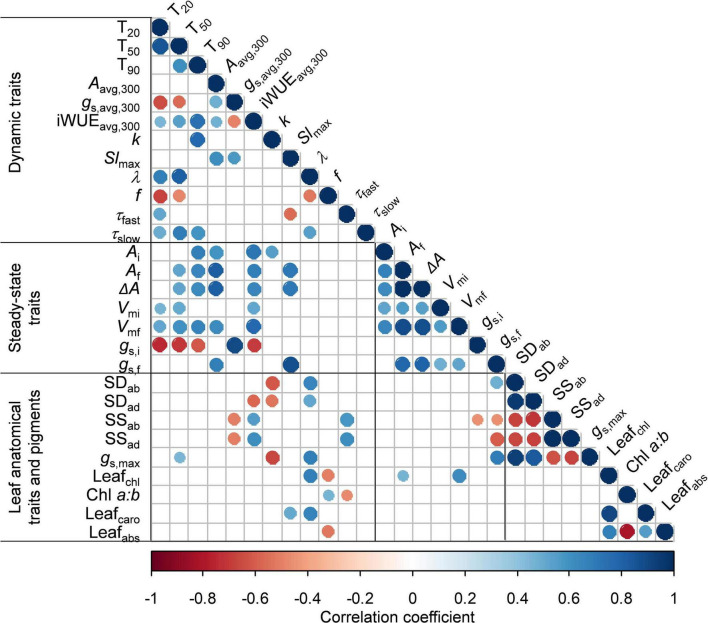
Correlation matrix among all traits measured in this study. Statistically significant (*p* < 0.05) negative linear correlations are shown in red, significant positive correlations are shown in blue, insignificant correlations are left blank. For definitions and units, refer to Table 2. Correlation coefficients and *p*-values for each correlation are given in Supplementary Table 3.

**FIGURE 8:**
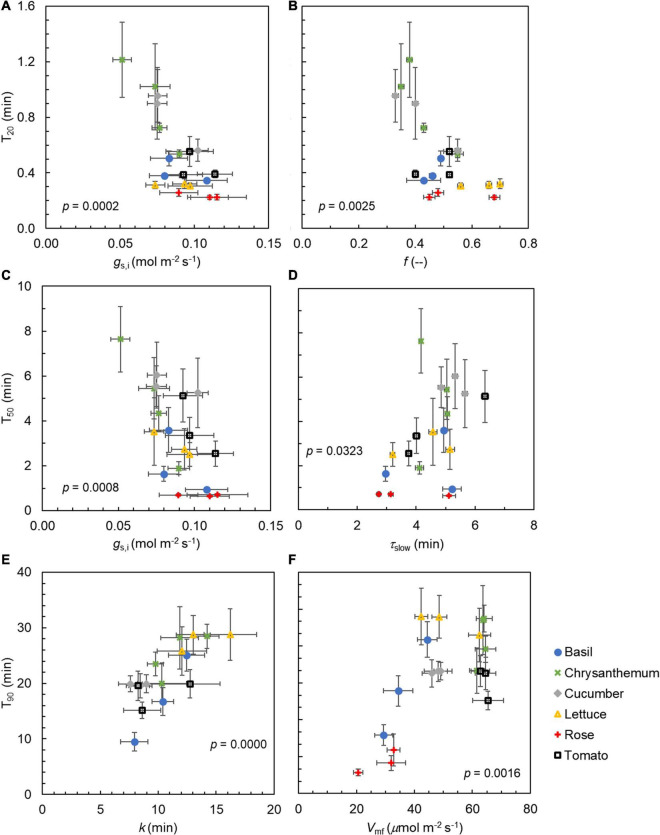
Relationships between **(A)** initial stomatal conductance in low irradiance (*g*_*s,i*_) and time to reach 20% of full photosynthetic induction (*T*_20_), **(B)** the weighting factor *f* describing the kinetics of maximum Rubisco carboxylation rate (*V*_cmax_) during photosynthetic induction and *T*_20_, **(C)** *g*_*s,i*_ and time to reach 50% of full photosynthetic induction (*T*_50_), **(D)** the time constant for the slow phase of *V*_cmax_ induction (*τ*_*slow*_) and *T*_50_, **(E)** time constant *k* for stomatal opening and time to reach 90% of full photosynthetic induction (*T*_90_), and **(F)** final *V*_cmax_ under 15 min of high irradiance (*V*_*mf*_) and *T*_90_. Datapoints are means ± SE (*n* = 6–9). Values shown are *p*-values of Pearson correlation. Data for rose is not presented in **(E)** due to absence of stomatal movement.

Generally, steady-state gas exchange traits correlated well with one another (e.g., *g*_*s,f*_ vs. *A*_*f*_, *V*_*mf*_ vs. *A*_*f*_), as did dynamic traits (e.g., *k* vs. T_90_; Figure 7). Some steady-state traits also correlated well with dynamic traits (e.g., *Sl*_max_ vs. *g*_*s,f*_; Figure 7). Importantly, we identified key traits that showed strong correlations with indicators of the rate of photosynthetic induction (i.e., *T*_20_, *T*_50,_ or *T*_90_); these key traits were relevant to either stomata and their rate of movement (*g*_*s,i*_ and *k*) or Rubisco activation (*f*, τ_*slow*_, and *V*_*mf*_) (Figure 8). Furthermore, these traits represented either dynamic (*f*, τ_*slow*_, or *k*) or steady-state traits (*g*_*s,i*_ or *V*_*mf*_), suggesting that both types of the trait were relevant for the rate of photosynthetic induction. The value *g*_*s,i*_ correlated negatively with *T*_20_ and *T*_50_ (Figures 8A,C). *T*_20_ was also correlated with *f*, and *T*_50_ was correlated with τ_*slow*_ (Figures 8B,D). Both *k* and *V*_*mf*_ were positively correlated with *T*_90_, and *k* and *T*_90_ showed an especially strong linear correlation (Figures 8E,F). Given the strong correlations between photosynthetic induction traits (*T*_20_, *T*_50_, and *T*_90_) and stomatal parameters (*g*_*s,i*_ or *k*), we further tested whether stomatal conductance-related parameters were correlated with traits characterizing stomatal anatomy (stomatal size and density). The stomatal size was not correlated with either *g*_*s,i*_, *k*, or *Sl*_max_, but was negatively correlated with stomatal density across species (except for chrysanthemum, which had large stomates; Supplementary Figures 7A–D). Interestingly, there was a very strong linear correlation between stomatal size on the abaxial side with that on the adaxial side of the leaf (Supplementary Figure 7E), with stomatal size on the adaxial leaf surface being ∼93% of the size on the abaxial leaf surface for all species except for rose.

## Discussion

### Large Variation in Induction Kinetics Exists in Major Horticultural Species

Increasing the rate of photosynthesis is expected to increase crop yields (Ort et al., 2015; Simkin et al., 2019). Although the harvested product for horticultural crops can be very different from staple food crops, e.g., fresh flowers, fruits, and flavor additives, biomass production (thus photosynthesis) remains the basis for high yield and good product quality. For example, flower number was positively correlated with plant dry weight in chrysanthemum (Carvalho and Heuvelink, 2003), and extra assimilates contributed by the canopy improved the stem quality of cut-rose (Zhang et al., 2020). Therefore, increasing photosynthesis is important for optimizing horticultural crop production, especially where growth in most crops can be assumed to be a source rather than sink limited for most of the production season (Marcelis, 1994; Li et al., 2015).

Natural genetic variation is an important resource for breeding. The genotypic variation of photosynthesis has been examined widely regarding its steady-state traits (Flood et al., 2011). However, steady-state photosynthesis does not provide an accurate representation of operating photosynthesis under fluctuating light (which often happens in the field and greenhouses, e.g., Supplementary Figure 1), given that time constants of induction/relaxation of photosynthesis reduce the time-integrated rate of carbon fixation (Kromdijk et al., 2016; Morales et al., 2018). Speeding up photosynthetic induction has been suggested as an important breeding target (Tanaka et al., 2019; Qu et al., 2020). Large genotypic variation of photosynthetic induction has been found in field crops (e.g., rice, wheat, soybean, and cassava) and woody species in forestry systems, proving that breeding for improving the dynamic crop photosynthesis is feasible (Valladares et al., 1997; Soleh et al., 2017; Salter et al., 2019; Acevedo-Siaca et al., 2020; De Souza et al., 2020). Here, we show that a large genotypic variation in photosynthetic induction also exists between major horticultural crops, and generally this variation for dynamic traits is larger than the variation for steady-state traits. Also, variation between crops in photosynthetic induction was generally larger than the variation between cultivars of the same crop.

Variation of photosynthetic induction in these 19 horticultural genotypes was quantified under near-optimal conditions, i.e., climate control management in the greenhouse was done similarly as in commercial greenhouse production. This is similar to other studies that aimed to quantify genotypic variation in crops, such as rice and cassava (De Souza et al., 2020; Acevedo-Siaca et al., 2021). However, abiotic stresses often occur during crop growth, not only in the field but also in low-tech greenhouses. When testing a genotypic variation of steady-state photosynthetic traits under both well-watered and drought conditions, drought accounted for a larger proportion of total variation compared with the genotypic variation (Gu et al., 2012). Genotypic variation of dynamic photosynthetic traits could potentially be coupled with variations induced by environmental fluctuations other than irradiance. For example, genotypic variation in intrinsic water-use efficiency found in our and other studies (Acevedo-Siaca et al., 2021) could lead to different crop performance between genotypes when drought occurs. Additionally, a recent study suggested that taking into account photosynthetic induction effects led to a reduction of 2–7% in the estimation of daily carbon gain (Murakami and Jishi, 2021), which is much smaller than the estimation error predicted by earlier studies (Naumburg and Ellsworth, 2002; Taylor and Long, 2017) and in real measurements (Adachi et al., 2019). The patterns of irradiance fluctuations appear to be very important in determining the discrepancy between simulating daily carbon gain with and without the effects of photosynthetic induction (Murakami and Jishi, 2021). However, only few studies quantified irradiance fluctuations in greenhouses at the relevant time scales (van Westreenen et al., 2020), hampering such estimations for the greenhouse production context. Moreover, previous irradiances potentially affect photosynthetic induction responses to the upcoming irradiance (Jackson et al., 1991; Kaiser et al., 2017). Further studies are needed to quantify the genotypic variation of dynamic photosynthesis under stress conditions and to evaluate their importance in different irradiance fluctuation patterns under greenhouse conditions with considering photosynthetic induction rates across different irradiances.

### Variation in Photosynthetic Induction of Horticultural Crops Is Mostly Driven by Differences in Stomatal Traits

Photosynthetic induction is mainly regulated by three transient limitations: RuBP regeneration, Rubisco activation, and stomatal opening (Pearcy, 1953). We found large (CV up to 68%, Table 2) genotypic variation in the kinetics of stomatal responses to an irradiance increase, compared to the genotypic variation in the other two limitations. A large variation in the stomatal opening time was also found across 15 vascular plants including fern, gymnosperm, and angiosperm species (Deans et al., 2019), indicating that strong genotypic variation of stomatal response kinetics exists in many species. In our study, *Sl*_max_ was 0–0.3 μmol m^–2^ s^–2^, and values for *k* varied between 8 and 16 min (Table 2). These values of Slmax and *k* are within the range of those found for other species that had partially grown and evolved outdoors (McAusland et al., 2016), suggesting that the specific indoor growth conditions horticultural crops experienced do not influence the rapidity of stomatal opening. Faster stomatal opening tends to speed up photosynthetic induction (Shimadzu et al., 2019; Yamori et al., 2020), and our results showed a strong linear correlation between *k* and *T*_90_ (Figure 8E), indicating that genotypes that require less time to open their stomata reach full photosynthetic induction faster. The strong correlation between the time constants of stomatal opening and the time to approach full photosynthetic induction also suggests that stomatal effects are typically the major ones left in the later phase of photosynthetic induction. Moreover, a higher initial *g*_s_ before an irradiance increase led to a faster speed of photosynthetic induction (Figures 8A,C), which is confirmatory of many previous studies (Soleh et al., 2017; Kaiser et al., 2020; Sakoda et al., 2020). These results highlight the importance of stomatal traits to explain the variations in photosynthetic induction, not only between genotypes of the same crop but also among different crops.

The speed of the stomatal response to environmental changes is generally considered to be related to the stomatal size (Hetherington and Woodward, 2003; Raven, 2014). A negative correlation exists between the stomatal size and the speed of *g*_s_ increase upon an irradiance increase, which has been found in many species (Drake et al., 2013; Kardiman and Ræbild, 2018). In addition, the relationship between average pore aperture and *g*_s_ is nonlinear (Kaiser and Kappen, 2000, 2001), which means that similar stomatal opening responses could result in different *g*_s_ kinetics, depending on the anatomical features of the stomatal complex in a given species. This nonlinear change in scale can also result in different time constants (e.g., time to reach 50% of the total variation) for the kinetic of pore aperture compared to *g*_s_. We found that the pore area of cucumber and tomato (which had relatively small stomates) tended to reach a plateau earlier after an irradiance increase than that in chrysanthemum (which had relatively large stomates) (Figures 5A, 6B,C). The time needed to reach 50% of the final pore area in high irradiance was found to be higher in chrysanthemum than in cucumber and tomato (Supplementary Figure 8), suggesting that horticultural species with larger stomates require more time to open their stomata. However, this does not necessarily lead to a close correlation between parameters of *g*_s_ kinetics (*k* and *Sl*_max_) and stomatal size (Supplementary Figures 7B,C). This could be due to the fact that stomatal density also determines *g*_s_, and the range of genotypic variation in these traits may also be too small to identify the correlations. Both tomato and cucumber showed large absolute changes in *g*_s_ for low- and high irradiance adapted leaves (Supplementary Figure 4). However, changes in absolute pore area for low- and high-irradiance adapted leaves in cucumber and tomato were rather small, compared to other crops (Figure 6C). This could result from relatively high stomatal density in cucumber and tomato (Figure 5B), magnifying small changes in an individual pore area. In the hypothetical situation of a cucumber leaf having a low stomatal density, such as that of chrysanthemum, stomata in this cucumber leaf would need to open their individual pore area up to ∼40 μm^2^ to achieve the observed increase in *g*_s_ (Supplementary Figure 9A). In contrast, the pore area of chrysanthemum substantially increased after exposure to high irradiance, but due to a low stomatal density, this did not lead to a large increase in *g*_s_ (Figures 5B, 6B and Supplementary Figure 4). When using the hypothetical situation of a chrysanthemum leaf possessing the stomatal density of a cucumber leaf, stomata in the chrysanthemum leaf only needed to open to a very small extent (∼3 μm^2^) to achieve the observed *g*_s_ increase (Supplementary Figure 9B). These results suggest that species having small but many stomates are more efficient in adjusting *g*_s_ to changes in irradiance, as it only requires small changes in individual pores to achieve large changes in *g*_s_.

Interestingly, the actual pore area opening generally accounted for less than 10% of the theoretical maximum pore area in all genotypes (Figures 6D–F), resulting in an average ratio between *g*_*s,f*_ and *g*_*s,max*_ (determined by anatomical traits) of 0.1 across genotypes (Supplementary Figure 10). This average *g*_*s,f*_/*g*_*s,max*_ ratio among horticultural crops is generally lower than what has been found in previous studies (McElwain et al., 2016; Murray et al., 2020). In a modeling study, Dow et al. (2014) predicted an optimal ratio between operating *g*_s_ and anatomical *g*_*s,max*_ of 0.2; their study suggested that at 20% operating capacity, guard cells could increase the pore size efficiently when favorable conditions persisted, but could also close the pore just as quickly under stress (Dow et al., 2014). While experimental studies on the *g*_s_-*g*_*s,max*_ relationship across species are scarce, some have described a relatively constant ratio of 0.25 between operating *g*_s_ and *g*_*s,max*_ in shrub and tree species (McElwain et al., 2016; Murray et al., 2020). Our results suggest that for horticultural crops, operating *g*_s_ at 10% of its maximum capacity may be already sufficient for guard cells to function efficiently.

### Variation in Biochemical Processes During Photosynthetic Induction Is Less Strong Than Differences in Stomatal Traits in Horticultural Crops

The initial, fast phase of photosynthetic induction involves the availability of RuBP and other Calvin cycle intermediates and is assumed to last 1–2 min (Pearcy, 1953; Sassenrath-Cole and Pearcy, 1992). The time constant for the fast phase of *V*_cmax_ induction (τ_*fast*_; Figure 4A) may indicate the speed of completing the initial phase of photosynthetic induction, but this assumption needs to be verified using Calvin cycle metabolomics studies. Here, τ_*fast*_ varied between 0.5 and 1.1 min, which is generally larger than what has been found across wheat cultivars (0.3–0.5 min; Salter et al., 2019). This may suggest higher activities and/or amounts of fructose-1,6-bisphosphatase (FBPase), sedoheptulose-1,7-bisphosphatase (SBPase), and phosphoribulokinase (PRK) in field agronomic crops than in horticultural crops, given that the activation of RuBP regeneration is mainly limited by these three enzymes (reviewed by Kaiser et al., 2018). Nevertheless, our results confirm that the time needed to complete the initial phase of photosynthetic induction is generally rapid, and this is especially true when the leaf was adapted to low irradiance instead of darkness before switching to a high irradiance (Kaiser et al., 2017), as was the case in this study.

The following, slow phase of photosynthetic induction involves light-dependent activation of Rubisco by Rubisco activase, and this phase seems to show more variation between species: time constants of 4–5 min were reported for *Alocasia macrorrhiza* and *Spinacia oleracea*, and 2–4 min for wheat (Pearcy, 1953; Salter et al., 2019). For the horticultural genotypes examined here, we found slightly larger time constants (τ_*slow*_) of 3–7 min (Figure 4B). The rate of Rubisco activation has been found to be an important determinant of photosynthetic induction in many species (e.g., wheat and soybean) (Soleh et al., 2017; Salter et al., 2019). However, we found a relatively smaller variation in Rubisco activation rate compared to variations found in many other traits. A CV of 22% was found for τ_*slow*_, which was less than the CV of photosynthetic induction (e.g., 61% for T_50_) and traits related to stomatal opening (e.g., 68% for *Sl*_max_; Table 2). This corresponds with the findings of Deans et al. (2019) who found that the biochemical activation response time (5–25 min) was much more conserved between species (including angiosperms, ferns, and gymnosperms) than the time required for stomatal opening (10–150 min). It is worth noting that although the dynamic *A* vs. *C*_*i*_ approach has been used in many studies to quantify *V*_cmax_ kinetics during photosynthetic induction (Soleh et al., 2016; Taylor and Long, 2017; Salter et al., 2019; De Souza et al., 2020), the original FvCB model describes steady-state photosynthesis. By applying the FvCB model on dynamic *A* vs. *C*_*i*_, it was assumed that the slow *A* induction changes are mainly caused by Rubisco activation. Although the role of Rubisco activation during *A* induction has been verified experimentally (Taylor et al., 2022), other processes, such as changes in mesophyll conductance could also play a role during *A* induction (Liu et al., 2021; Sakoda et al., 2021). However, mesophyll conductance changes have been suggested to be far more rapid than the observed *V*_cmax_ kinetics presented here, and the relative importance of mesophyll conductance for *A* induction is still under debate (De Souza et al., 2020; Liu et al., 2021; Sakoda et al., 2021). We conclude that the variation in Rubisco activation kinetics among the six horticultural crops may not be the primary cause for the large variation found in photosynthetic induction.

In some species (chrysanthemum, lettuce, and tomato), photosynthetic induction in the first 1–2 min exhibited a transient drop when photosynthetic induction was measured under high CO_2_ (>600 ppm; Supplementary Figure 11). This is likely caused by a limited amount of inorganic phosphate (Pi) in the metabolite pool of the Calvin cycle, due to insufficient and slow activation of sucrose-phosphate synthase (SPS) during the initial phase of the light increase (Stitt and Quick, 1989; Huber and Huber, 1992). Supposedly, during the first 1–2 min of the irradiance increase, the amount of free Pi is sufficient to support photosynthesis independently of any end-product synthesis. However, once Pi is exhausted, photosynthesis is inhibited until the conversion of triose-phosphates to sucrose in the cytosol releases enough Pi, which can then be translocated back into the chloroplast (Stitt and Quick, 1989). The activation of SPS is regulated by irradiance in some species (e.g., barley and maize) but not in others (e.g., soybean, tobacco, and cucumber) (Huber et al., 1989), leading to species variations in the level of Pi limitation. This may explain why in our results, the transient drop of photosynthesis in high CO_2_ was seen in some species only (Supplementary Figure 11).

### Implications for Horticultural Crop Breeding

We showed that in major horticultural crops, transient limitations to photosynthetic induction appeared to be species-dependent, but the general trend was that there was a large genotypic variation in the level of transient stomatal limitation, whereas the extent of transient non-stomatal limitation during photosynthetic induction was relatively conserved (Supplementary Figures 5C,D). Previous studies showed that in rice, the primary transient limitation was biochemical, whereas, in cassava, primary limitations were caused by stomata (Yamori et al., 2012; De Souza et al., 2020). For horticultural species, photosynthesis transients of some crops (e.g., cucumber) tended to be limited by biochemistry and stomata to a comparative extent, whereas those in other crops (e.g., lettuce and chrysanthemum) tended to be more strongly limited by stomata (Supplementary Figures 5C,D). Stomatal size may partially regulate the level of stomatal limitation during photosynthesis induction. Species (e.g., rice) with smaller stomata have been found to show a low level of stomatal limitation (Acevedo-Siaca et al., 2020). In our study, chrysanthemum, which had the largest stomata among the tested greenhouse crops, showed the highest level of transient stomatal limitation (Figures 3C, 5A and Supplementary Figure 5D). This is possibly due to the fact that larger stomata need more time to open until a new steady state has reached (Drake et al., 2013; also refer to Figures 6A–C and Supplementary Figure 8), resulting in a higher level of transient stomatal limitation during photosynthetic induction.

Species with small stomata displayed high stomatal density, which in the case of incomplete stomatal closure may lead to high transpiration and increased water demand (e.g., during the night). Reduced stomatal density improves drought tolerance in species, such as rice and barley (Hughes et al., 2017; Caine et al., 2019). We found that the two cut-flowers have relatively low total stomatal density (including both leaf surfaces), which possibly favors vase life by increasing water conservation, such as in other cut-flowers (e.g., *Antirrhinum majus* L., Schroeder and Stimart, 2005). Altogether, these results suggest that manipulating stomatal traits rather than biochemical traits is more relevant for horticultural crop breeding.

Additionally, we found a highly conserved ratio between stomatal size at the abaxial and adaxial leaf surface, as well as between the stomatal densities on both leaf sides in all crops, except for rose (Supplementary Figures 7E,F). Stomatal size and density at the adaxial leaf surface were respectively 93 and 71% of the size and density at the abaxial leaf surface. A linear correlation between the stomatal densities of both leaf sides has previously been found in rice and tomato, with more stomata on the abaxial leaf surface (Fanourakis et al., 2015; Zhang et al., 2019). The distribution of stomatal density between the two leaf sides is relevant for total *g*_s_ partitioning between leaf sides (Ticha, 1982). A more uniform *g*_s_ partitioning favors CO_2_ diffusion inside the leaf, and therefore gas exchange (Parkhurst and Mott, 1990; Muir et al., 2014), but may come at the expense of stress resilience in the field. Milla et al. (2013) found that wild species showed a larger difference in stomatal density between leaf sides, while domestication tended to reduce the difference of stomatal density between leaf sides, by lowering the stomatal density at the abaxial side. Interestingly, wild but not domesticated tomato genotypes showed even stomatal distribution between leaf sides (Koenig et al., 2013; Fanourakis et al., 2015). Given the potential effects of *g*_s_ partitioning between leaf sides on gas exchange, further studies are needed to explore whether or not a uniform *g*_s_ partitioning favors photosynthetic induction and the underlying mechanisms that regulate the distribution of stomatal density between leaf sides for breeding.

## Conclusion

Large variations in the rate of photosynthetic induction were found among 19 genotypes from six of the world’s most commercially relevant horticultural crops. Variations in stomatal density and size and their effects on dynamic changes in the stomatal conductance were the major determinants of variation in the rate of photosynthetic induction, not only between crops but also between cultivars of the same crop. RuBP regeneration and Rubisco activation during photosynthetic induction exhibited relatively less genotypic variation (CV up to 22%) than did stomatal traits (CV up to 68%). Crops with large but few stomata tended to have a slow increase in stomatal conductance, potentially leading to a high level of transient stomatal limitation during photosynthetic induction. All horticultural genotypes showed an operational *g*_s_ of ∼10% of its maximum capacity, which was lower than the average *g*_s_/*g*_*s,max*_ ratio found in previous studies. The ratio of stomatal size between abaxial and adaxial leaf surfaces was highly conserved among horticultural crops, as was the ratio of stomatal density, suggesting that the partitioning of *g*_s_ between leaf surfaces was hardly affected by species difference when under similar growth conditions. Our results highlight the importance of manipulating stomatal traits for speeding up photosynthetic induction in horticultural crops.

## Data Availability Statement

The original contributions presented in the study are included in the article/Supplementary Material, further inquiries can be directed to the corresponding author/s.

## Author Contributions

NZ, LM, and EK designed the research. NZ and SB conducted the measurements. NZ, SB, and DJ analyzed the data, with suggestions from SV-C and EK. EK and LM secured funding. NZ drafted the manuscript. SB, DJ, SV-C, LM, and EK made substantial contributions to improve the manuscript. All authors contributed to the article and approved the submitted version.

## Conflict of Interest

The authors declare that the research was conducted in the absence of any commercial or financial relationships that could be construed as a potential conflict of interest.

## Publisher’s Note

All claims expressed in this article are solely those of the authors and do not necessarily represent those of their affiliated organizations, or those of the publisher, the editors and the reviewers. Any product that may be evaluated in this article, or claim that may be made by its manufacturer, is not guaranteed or endorsed by the publisher.

## Footnotes

1
http://www.r-project.org/

## Supplementary Material

Supplementary Table 1 Ion concentrations of the nutrient solution.

Supplementary Table 2 Stomatal pore length and guard cell width.

Supplementary Table 3 Correlation coefficients and *p*-values for all focused traits (provided as a separate excel file).

Supplementary Figure 1 Irradiance fluctuations measured in the greenhouse.

Supplementary Figure 2 Distribution of greenhouse supplemental light.

Supplementary Figure 3 Greenhouse climate conditions during the experiment.

Supplementary Figure 4 Final stomatal conductance reached at low and high irradiance.

Supplementary Figure 5 Dynamics of Rubisco carboxylation rate, electron transport rate, and transient nonstomatal and stomatal limitations during photosynthetic induction.

Supplementary Figure 6 Leaf pigment contents and light absorptance.

Supplementary Figure 7 Relationships between stomatal anatomical traits vs. parameters of stomatal conductance kinetics.

Supplementary Figure 8 Time needed to reach 50% of the final pore area in high irradiance.

Supplementary Figure 9 Kinetics of pore area opening of cucumber and chrysanthemum.

Supplementary Figure 10 Ratio between the operating and maximum stomatal conductance.

Supplementary Figure 11 Photosynthetic induction curves measured at different [CO_2_].

Supplementary Method 1 Description of the FvCB model.

Supplementary Method 2 Steps to solve the equation for stomatal pore area.

Supplementary Presentation 1 Comparisons between the measured and predicted leaf net photosynthesis rates.
