# Corrigendum: Variation of photosynthetic induction in major horticultural crops is mostly driven by differences in stomatal traits

**DOI:** 10.3389/fpls.2023.1252101

**Published:** 2023-08-02

**Authors:** Ningyi Zhang, Sarah R. Berman, Dominique Joubert, Silvere Vialet-Chabrand, Leo F. M. Marcelis, Elias Kaiser

**Affiliations:** ^1^ Horticulture and Product Physiology, Department of Plant Sciences, Wageningen University & Research, Wageningen, Netherlands; ^2^ Biometris, Department of Mathematical and Statistical Methods, Wageningen University & Research, Wageningen, Netherlands

**Keywords:** induction, genotypic variation, light fluctuations, modeling, photosynthesis, Rubisco activation, stomatal opening

In the published article, there was an error in **Table 2** as published. Stomatal size should have been calculated as π × stomatal length × stomatal width/4, therefore, all stomatal size values reported in this table (i.e. SS_ab_ and SS_ad_) should have been divided by four. The corrected **Table 2** and its caption “Definition, unit, maximum, minimum, mean and coefficient of variation (CV) for dynamic, steady-state, anatomical and physiological traits across 19 horticultural genotypes. Maximum and minimum values are average values of 6-9 replicates” appears below.

**Table 2:** Definition, unit, maximum, minimum, mean and coefficient of variation (CV) for dynamic, steady-state, anatomical and physiological traits across 19 horticultural genotypes.

Trait	Definition	Unit	Max. (genotype)	Min. (genotype)	Mean	CV (%)
Dynamic traits						
T_20_	Time to reach 20% of full *A* induction	min	1.2 (CHB)	0.2 (RAP)	0.5	55
T_50_	Time to reach 50% of full *A* induction	min	7.6 (CHB)	0.6 (RAP)	3.4	61
T_90_	Time to reach 90% of full *A* induction	min	28.8 (LGI)	3.4 (RAP)	19.2	42
*A*_avg,300_	Average *A* during the first 300 s of induction	*μ*mol m^-2^ s^-1^	10.9 (TB)	4.7 (RAP)	7.7	22
*g*_s,avg,300_	Average *g*_s_ during the first 300 s of induction	mol m^-2^ s^-1^	0.143 (TS)	0.052 (CHB)	0.099	22
iWUE_avg,300_	Average intrinsic water-use efficiency during the first 300 s of induction (*A*_avg,300_/*g*_s,avg,300_)	*μ*mol CO_2_ (mol H_2_O)^-1^	117 (CHB)	42 (RAP)	84	21
*k*	Time constant for *g*_s_ response to irradiance change ^1^	min	16.2 (LGI)	7.6 (CUH)	10.8	23
*Sl*_max_	Maximum rate of *g*_s_ response to irradiance change ^1^	mmol m^-2^ s^-2^	0.28 (CUH)	0.03 (CHA)	0.13	68
*λ*	Initial time lag of *g*_s_ response to irradiance change ^1^	min	7.4 (CUP)	0.1 (BR)	3.9	62
*f*	Weighting factor (between 0-1) for the fast and slow phase of *V*_cmax_ induction	–	0.7 (LGA)	0.4 (CHY)	0.5	18
*τ*_fast_	Time constant for fast phase of maximum Rubisco carboxylation rate (*V*_cmax_) induction	min	1.1 (CHA)	0.5 (LC)	0.7	22
*τ*_slow_	Time constant for slow phase of *V*_cmax_ induction	min	6.5 (TM)	3.1 (RAV)	4.8	22
Steady-state traits						
*A*_i_	Steady-state *A* at low irradiance	*μ*mol m^-2^ s^-1^	2.2 (TS)	0.7 (BR)	1.9	21
*A*_f_	Steady-state *A* at high irradiance	*μ*mol m^-2^ s^-1^	20.8 (TM)	5.7 (RAP)	14.4	30
Δ*A*	Difference between *A*_f_ and *A*_i_	*μ*mol m^-2^ s^-1^	18.8 (TM)	4.5 (RAP)	12.5	33
*V*_mi_	*V*_cmax_ at the start of photosynthetic induction	*μ*mol m^-2^ s^-1^	8.6 (CUP)	4.9 (BR)	7.0	16
*V*_mf_	*V*_cmax_ 15 min after start of photosynthetic induction	*μ*mol m^-2^ s^-1^	65.9 (TB)	20.6 (RAP)	49.9	29
*g*_s,i_	Steady-state *g*_s_ at low irradiance	mol m^-2^ s^-1^	0.12 (RRN)	0.05 (CHB)	0.09	19
*g*_s,f_	Steady-state *g*_s_ at high irradiance	mol m^-2^ s^-1^	0.51 (TS)	0.10 (RAV)	0.25	46
Leaf anatomical traits and pigments						
SD_ab_	Stomatal density at abaxial leaf side	mm^-2^	340 (CUP)	40 (LGA)	124	78
SD_ad_	Stomatal density at adaxial leaf side	mm^-2^	267 (CUH)	0 (RAP, RAV, RRN) ^2^	67	133
SS_ab_	Stomatal size at abaxial leaf side	*μ*m^2^	1411 (CHB)	210 (CUP)	681	57
SS_ad_	Stomatal size at adaxial leaf side	*μ*m^2^	1325 (CHR)	0 (RAP, RAV, RRN) ^2^	540	81
*g*_s,max_	Theoretical maximum *g*_s_, if all stomates were to open to their maximum extent	mol m^-2^ s^-1^	5.0 (CUP)	1.3 (LGI)	2.5	50
Leaf_chl_	Leaf chlorophyll content ^3^	mg m^-2^	222.0 (TM)	78.3 (LGA)	151.6	29
Chl *a*:*b*	Ratio of chlorophyll *a* to chlorophyll *b*	–	3.1 (LGA)	2.3 (BR)	2.7	7
Leaf_caro_	Leaf carotenoid content	mg m^-2^	28.4 (TM)	11.8 (BR)	19.1	25
Leaf_abs_	Leaf light absorptance ^4^	–	0.89 (BR)	0.73 (LGA)	0.82	5

Maximum and minimum values are average values of 6-9 replicates.

In the published article, there was an error in **Figure 5** as published. Stomatal size should have been calculated as π × stomatal length × stomatal width/4, therefore, the numbers shown in **Figure 5A** should have been divided by four. The corrected **Figure 5** and its caption “Stomatal size (A; SS_ab_) and density (B; SD_ab_) at the abaxial leaf side, and theoretical maximum stomatal conductance (C; *g*_s,max_) of all 19 horticultural genotypes. Colours indicate crop species. Bars show means ± s.e. (n = 7-9). Letters indicate significant differences (p < 0.05). Statistical test results of SS_ab_, SD_ab_ and *g*_s,max_ were based on log transformation of the data. See Table 1 for full genotype names” appears below.

**Figure 5:**
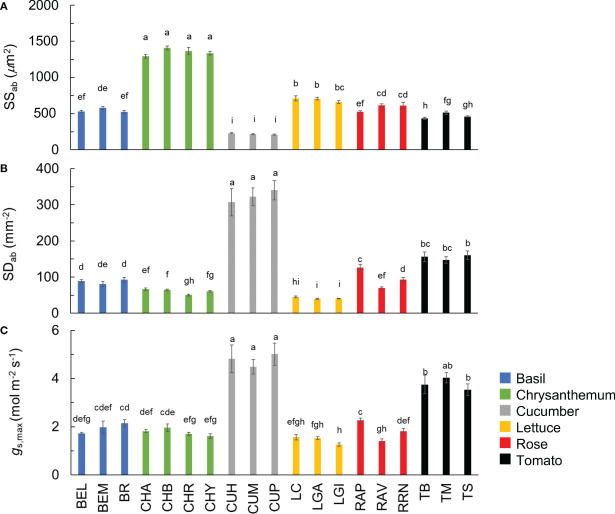
Stomatal size (**A**; SS_ab_) and density (**B**; SD_ab_) at the abaxial leaf side, and theoretical maximum stomatal conductance (**C**; *g*_s,max_) of all 19 horticultural genotypes. Colours indicate crop species. Bars show means ± s.e. (n = 7-9). Letters indicate significant differences (*p* < 0.05). Statistical test results of SS_ab_, SD_ab_ and *g*_s,max_ were based on log transformation of the data. See Table 1 for full genotype names.

The authors apologize for this error and state that this does not change the scientific conclusions of the article in any way. The original article has been updated.

In the published article, there was an error in Supplementary Data Sheet 1. All numbers along the x-axis of Figure S7A-E and along the y-axis of Figure S7E (i.e. stomatal size, which should have been calculated as π × stomatal length × stomatal width/4) in Supplementary Data Sheet 1 should have been divided by four. The correct material statement appears below.

