# Supplementary material for: Variation of Photosynthetic Induction in Major Horticultural Crops Is Mostly Driven by Differences in Stomatal Traits
Source: Front Plant Sci. 2022 Apr 27;13:860229. doi: 10.3389/fpls.2022.860229 (PMC9094112; doi:10.3389/fpls.2022.860229)
Supplement: Supplementary Table 1 — Ion concentrations of the nutrient solution. [file Data_Sheet_1.docx]

**Supplementary tables**

Table S1. Ion concentrations of the nutrient solution used in the experiment.

| Ion | Concentration | Unit |
| --- | --- | --- |
| NH_4_ | 0.6 | mmol L^-1^ |
| K | 6.8 | mmol L^-1^ |
| Na | 0.4 | mmol L^-1^ |
| Ca | 4.1 | mmol L^-1^ |
| Mg | 1.4 | mmol L^-1^ |
| NO_3_ | 12.4 | mmol L^-1^ |
| Cl | 0.3 | mmol L^-1^ |
| S | 2.3 | mmol L^-1^ |
| HCO_3_ | 0.8 | mmol L^-1^ |
| Si | 0.1 | mmol L^-1^ |
| P | 1.1 | mmol L^-1^ |
| Fe | 26 | *μ*mol L^-1^ |
| Mn | 9.8 | *μ*mol L^-1^ |
| Zn | 6.1 | *μ*mol L^-1^ |
| B | 37 | *μ*mol L^-1^ |
| Cu | 0.6 | *μ*mol L^-1^ |
| Mo | 0.7 | *μ*mol L^-1^ |

Table S2. Stomatal pore length and guard cell width at the abaxial leaf side of the 19 genotypes. Values are means ± s.e. (n = 7-9). See Table 1 for full genotype names.

| Genotype | Pore length (*μ*m) | Guard cell width (*μ*m) |
| --- | --- | --- |
| BEL | 18.4 ± 0.5 | 7.6 ± 0.2 |
| BEM | 21.5 ± 1.4 | 7.7 ± 0.3 |
| BR | 21.2 ± 0.5 | 7.5 ± 0.2 |
| CHA | 32.9 ± 1.3 | 12.1 ± 0.3 |
| CHB | 37.1 ± 2.3 | 12.2 ± 0.2 |
| CHR | 38.9 ± 0.7 | 11.7 ± 0.3 |
| CHY | 34.0 ± 1.1 | 12.2 ± 0.2 |
| CUH | 12.5 ± 0.3 | 5.2 ± 0.2 |
| CUM | 11.8 ± 0.4 | 5.0 ± 0.1 |
| CUP | 12.3 ± 0.4 | 5.0 ± 0.2 |
| LC | 22.2 ± 0.6 | 6.8 ± 0.3 |
| LGA | 24.1 ± 0.7 | 6.5 ± 0.3 |
| LGI | 24.1 ± 0.3 | 6.6 ± 0.3 |
| RAP | 23.3 ± 0.4 | 5.8 ± 0.3 |
| RAV | 25.8 ± 0.7 | 6.4 ± 0.3 |
| RRN | 24.9 ± 1.3 | 5.6 ± 0.2 |
| TB | 21.4 ± 0.6 | 6.0 ± 0.3 |
| TM | 25.5 ± 0.8 | 6.6 ± 0.2 |
| TS | 21.3 ± 0.6 | 6.4 ± 0.2 |

**Supplementary figures**


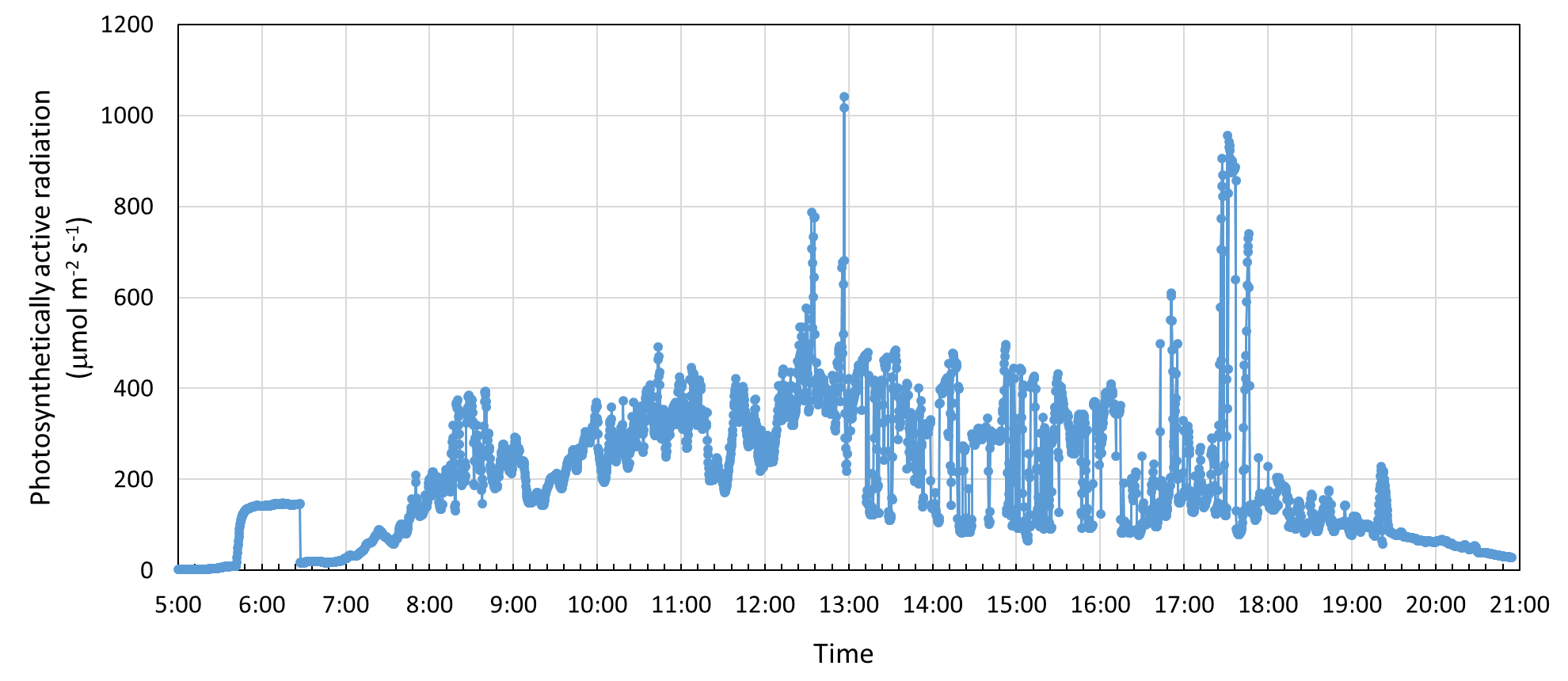


Figure S1. Photosynthetically active radiation (PAR) measured inside a greenhouse compartment (8 × 8 m) located in Wageningen, the Netherlands (52 °N, 6 °E). The height of the PAR sensor was 180 cm from the floor. Instantaneous PAR data were logged every 15 s between 5:00 and 21:00 on June 12^th^, 2021.

Figure S2. Heatmaps of distribution of supplemental light in the greenhouse compartment measured at (A) 90 cm (canopy level), (B) 70 cm and (C) 50 cm above the growth table. 0 cm represents the center of the compartment. Positive distances are distances from the center to the right (in *x*-axis direction) or front (*y*-axis direction) of the compartment. Negative distances are distances from the center to the left or the back of the compartment. Measurements were conducted after sunset.


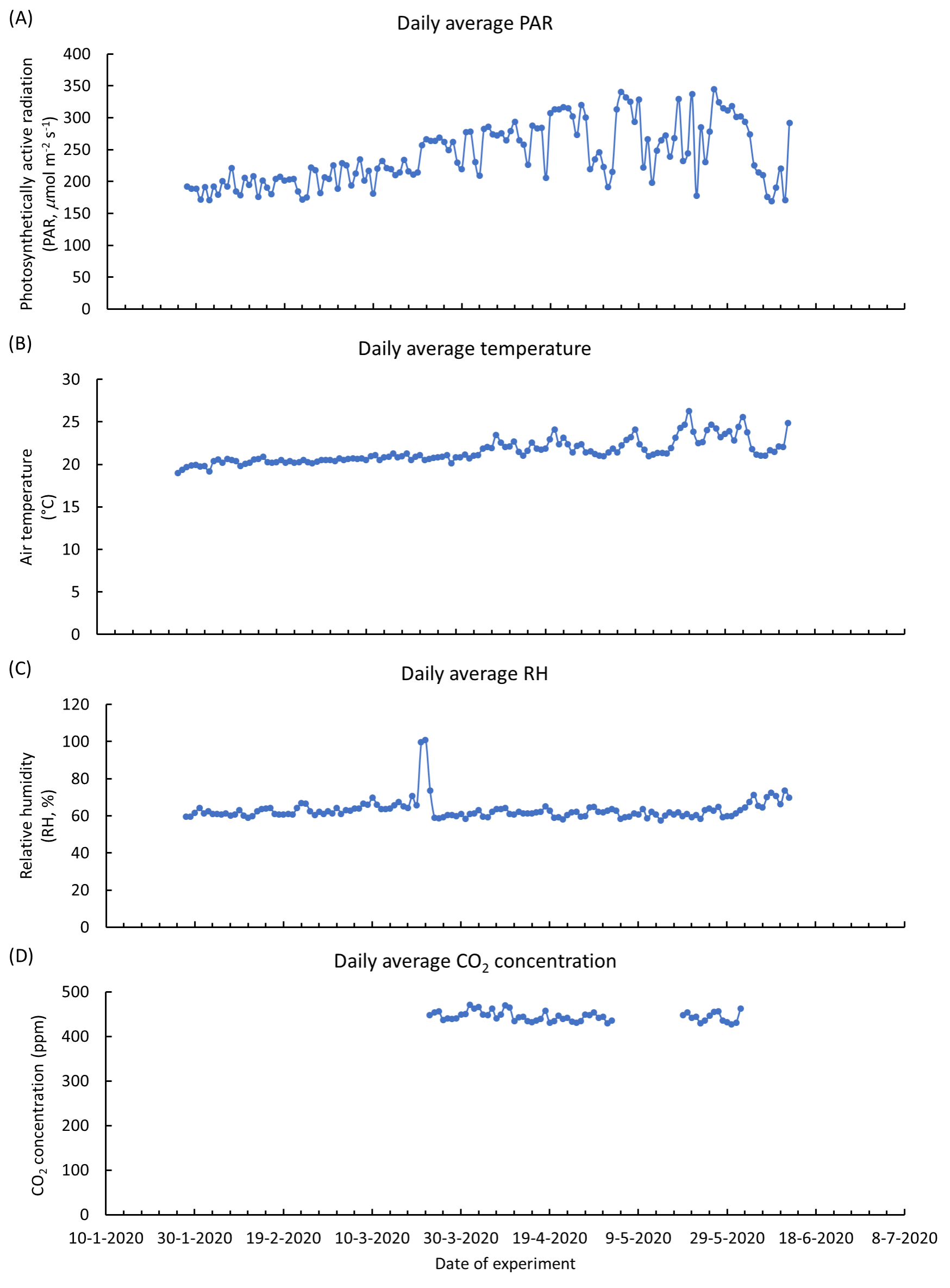


Figure S3. Daily average photosynthetically active radiation from both sunlight and assimilation lamps (A), air temperature (B), relative humidity (C) and [CO_2_] (D) inside the greenhouse compartment during the experiment. In panel D, recording of [CO_2_] data started from March 23^th^. Missing values (May 4^th^-18^th^) were due to sensor failure.


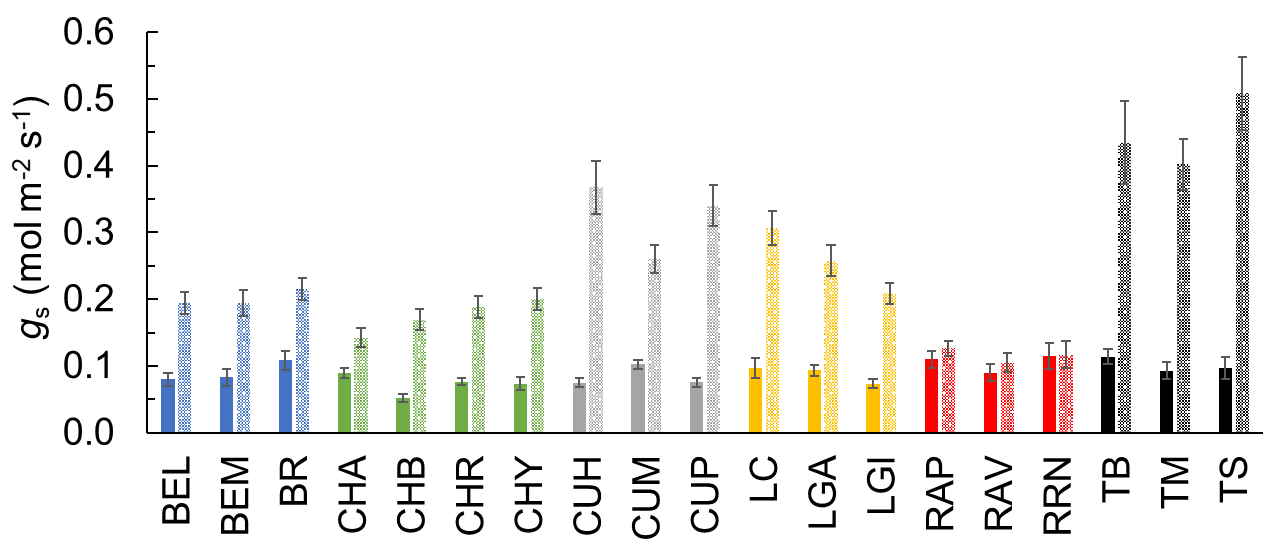


Figure S4. Steady-state stomatal conductance (*g*_s_) reached at low (solid bars) and high (shaded bars) irradiance in all 19 horticultural genotypes. Different colours indicate crop species. Bars show means ± s.e. (n = 7-9). See Table 1 for full genotype names.


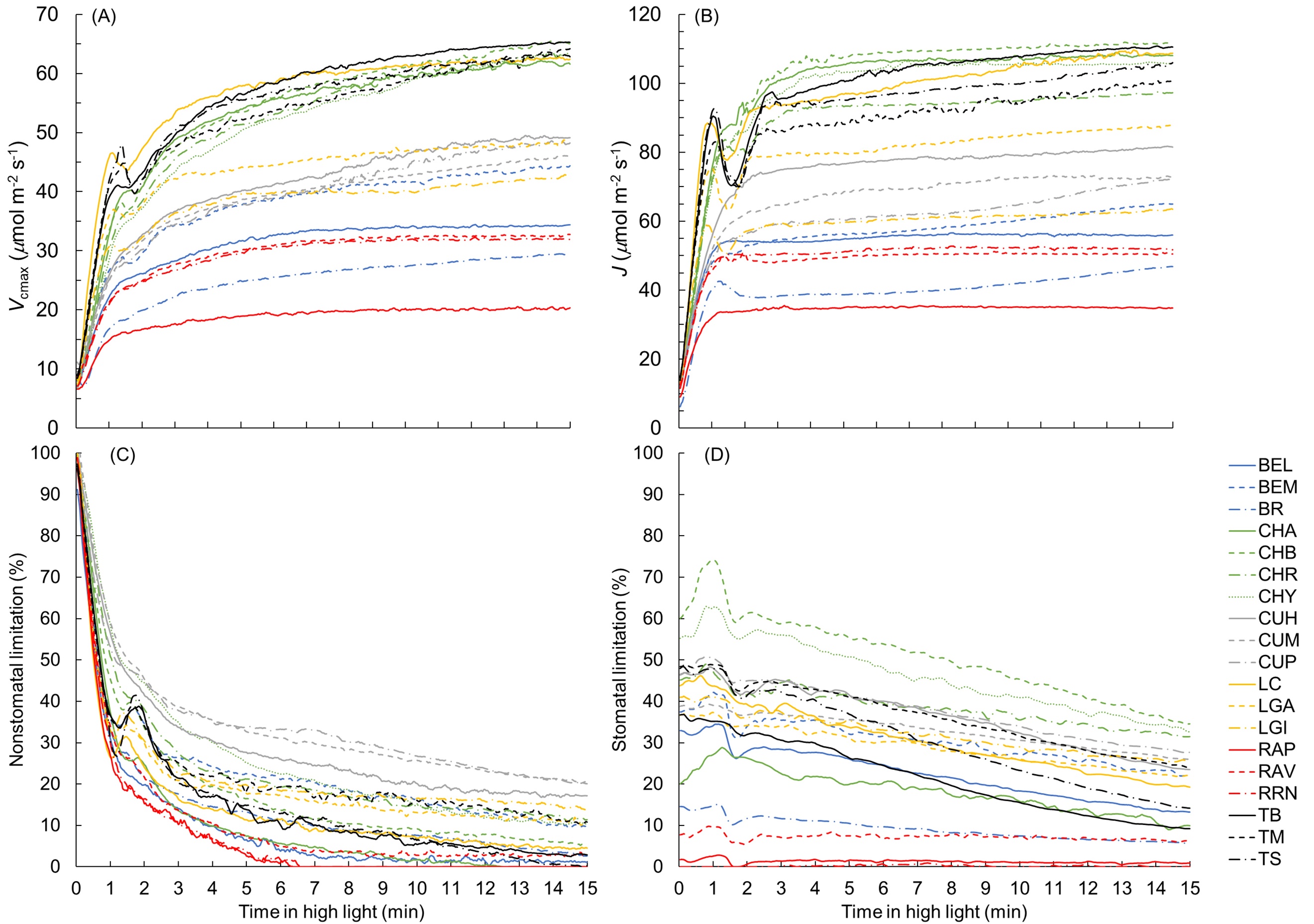


Figure S5. Dynamics of maximum Rubisco carboxylation rate *V*_cmax_ (A), electron transport rate *J* (B), as well as transient nonstomatal (C) and stomatal (D) limitations for photosynthesis during photosynthetic induction. Each curve represents the mean of 6-7 plants; s.e. of *V*_cmax_ was in the range 0.1-8.2 μmol m^-2^ s^-1^; s.e. of *J* was 0.2-9.0 μmol m^-2^ s^-1^; s.e. of nonstomatal limitation was 0.4-9.7%; s.e. of stomatal limitation was 1.2-8.1%; error bars omitted for greater visibility. See Table 1 for full genotype names.


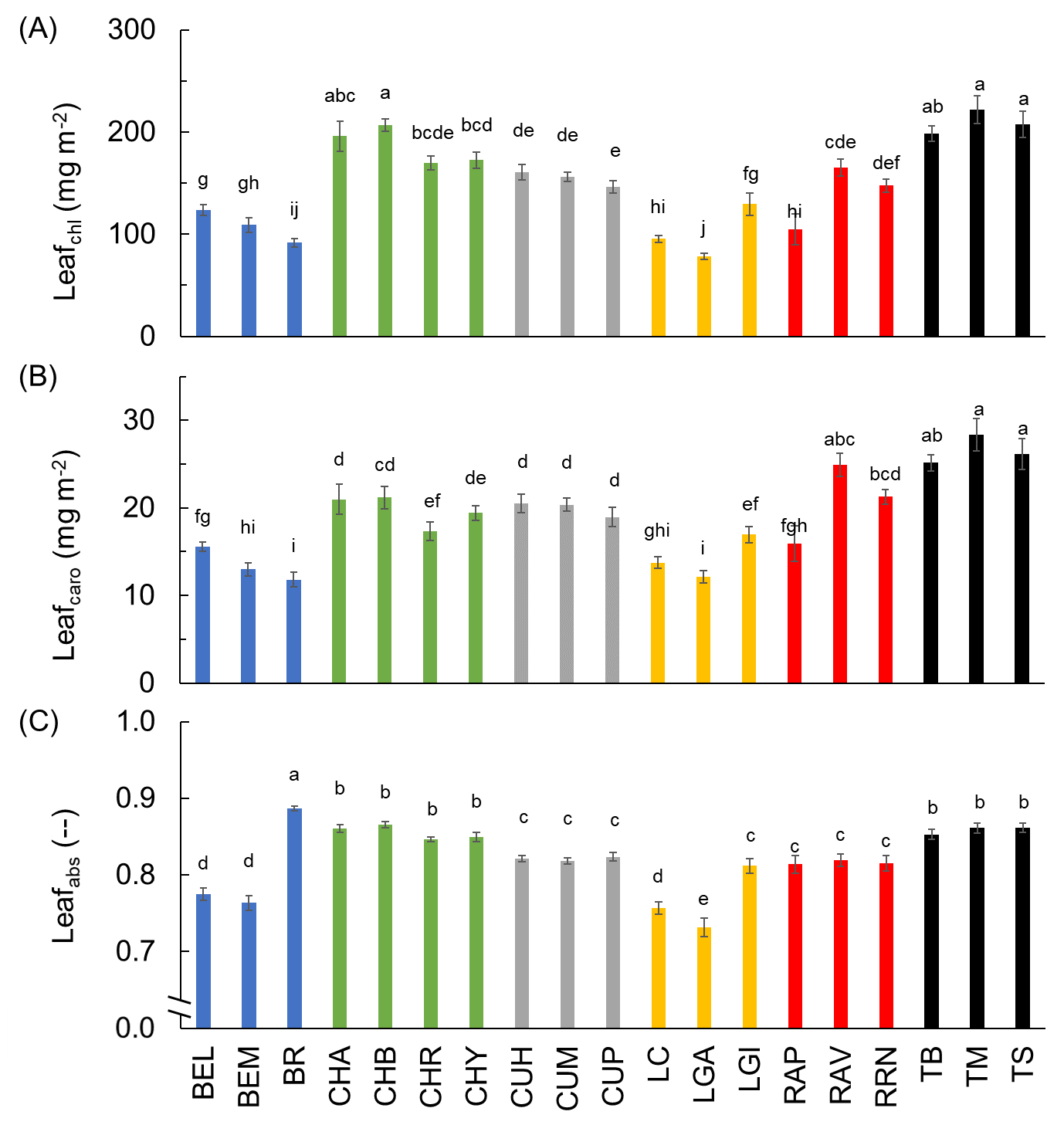


Figure S6. Leaf chlorophyll content (A), leaf carotenoid content (B) and leaf light absorptance in all 19 horticultural genotypes. Different colours indicate crop species. Bars show means ± s.e. (n = 7-9). Letters indicate significant differences (*p* < 0.05). Results of the statistical test for Leaf_chl_ and Leaf_caro_ were based on log transformation of data. See Table 1 for full genotypes names.

Figure S7. Relationships between stomatal size on the abaxial leaf side (SS_ab_) with initial stomatal conductance *g*_s,i_ (A), the time constant of stomatal opening *k* (B), maximum rate of stomatal response to irradiance change *Sl*_max_ (C), stomatal density at abaxial leaf side SD_ab_ (D) and stomatal size at adaxial leaf side SS_ad_ (E), and the relationship between SD_ab_ and stomatal density at adaxial leaf side (SD_ad_) (F). Datapoints are means ± s.e. (n = 7-9). Values shown are *p*-values of Pearson correlation. Data for rose not shown in (B) and (C) due to absence of stomatal opening, and not shown in (E) and (F) due to absence of stomata at leaf adaxial side.


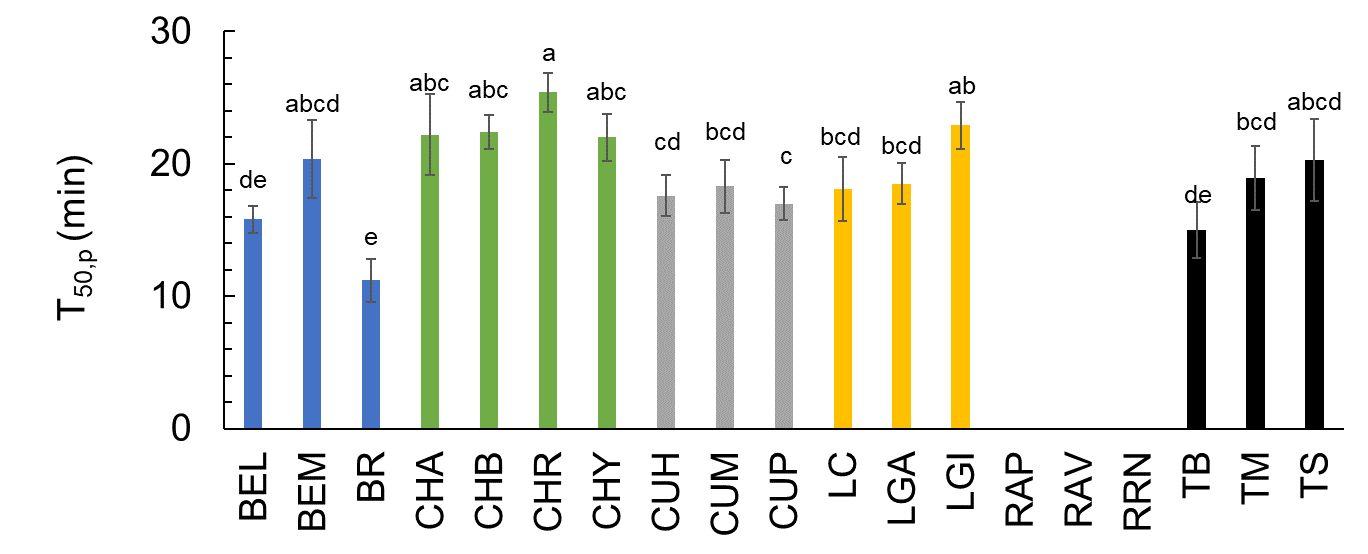


Figure S8. Time needed to reach 50% of the final pore area that can be reached in high irradiance (T_50,p_) in 16 horticultural genotypes (data from rose excluded). Different colours indicate crop species. Bars show means ± s.e. (n = 7-9). Letters indicate significant differences (*p* < 0.05). See Table 1 for full names of all genotypes.


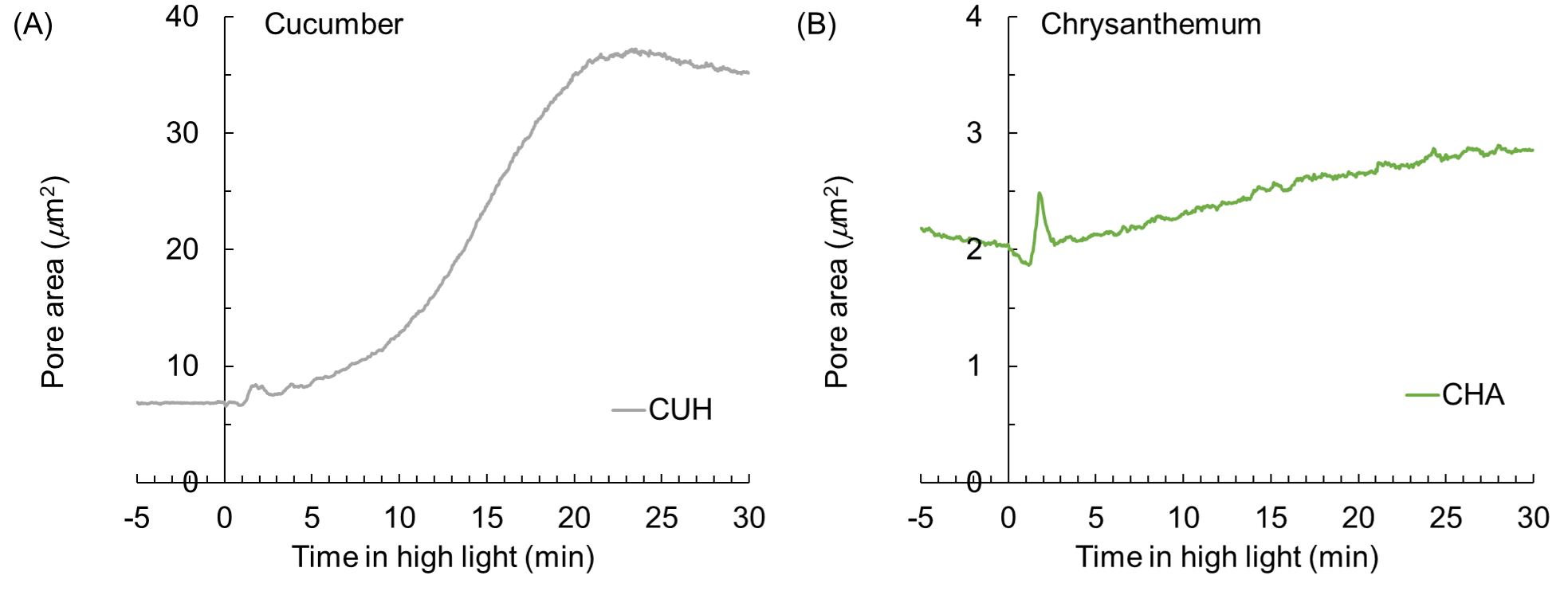


Figure S9. Kinetics of pore area opening of cucumber calculated using the stomatal density of chrysanthemum (A) and kinetics of pore area opening of chrysanthemum calculated using the stomatal density of cucumber (B) in response to a single-step change in irradiance. Time zero indicates the moment when irradiance was increased from 50 to 1000 *μ*mol m^-2^ s^-1^. See Table 1 for full genotype names.


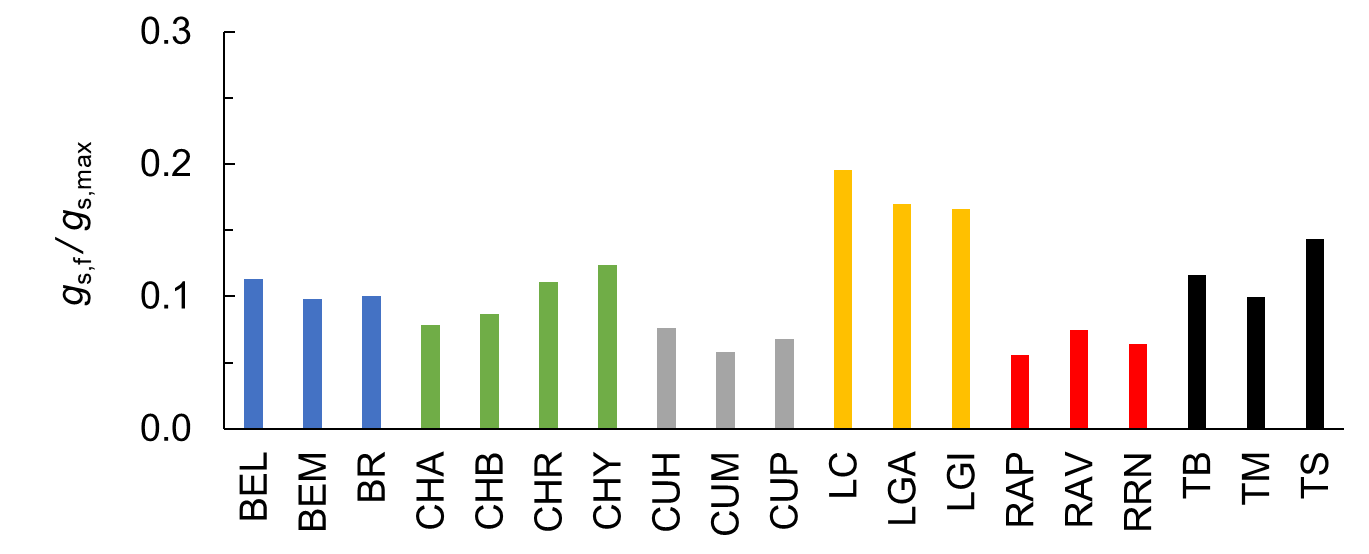


Figure S10. Ratio between steady-state stomatal conductance reached in high light (*g*_s,f_; see Fig. S4) and theoretical maximum stomatal conductance calculated based on stomatal anatomical data (*g*_s,max_; see Fig. 5) in 19 horticultural genotypes.


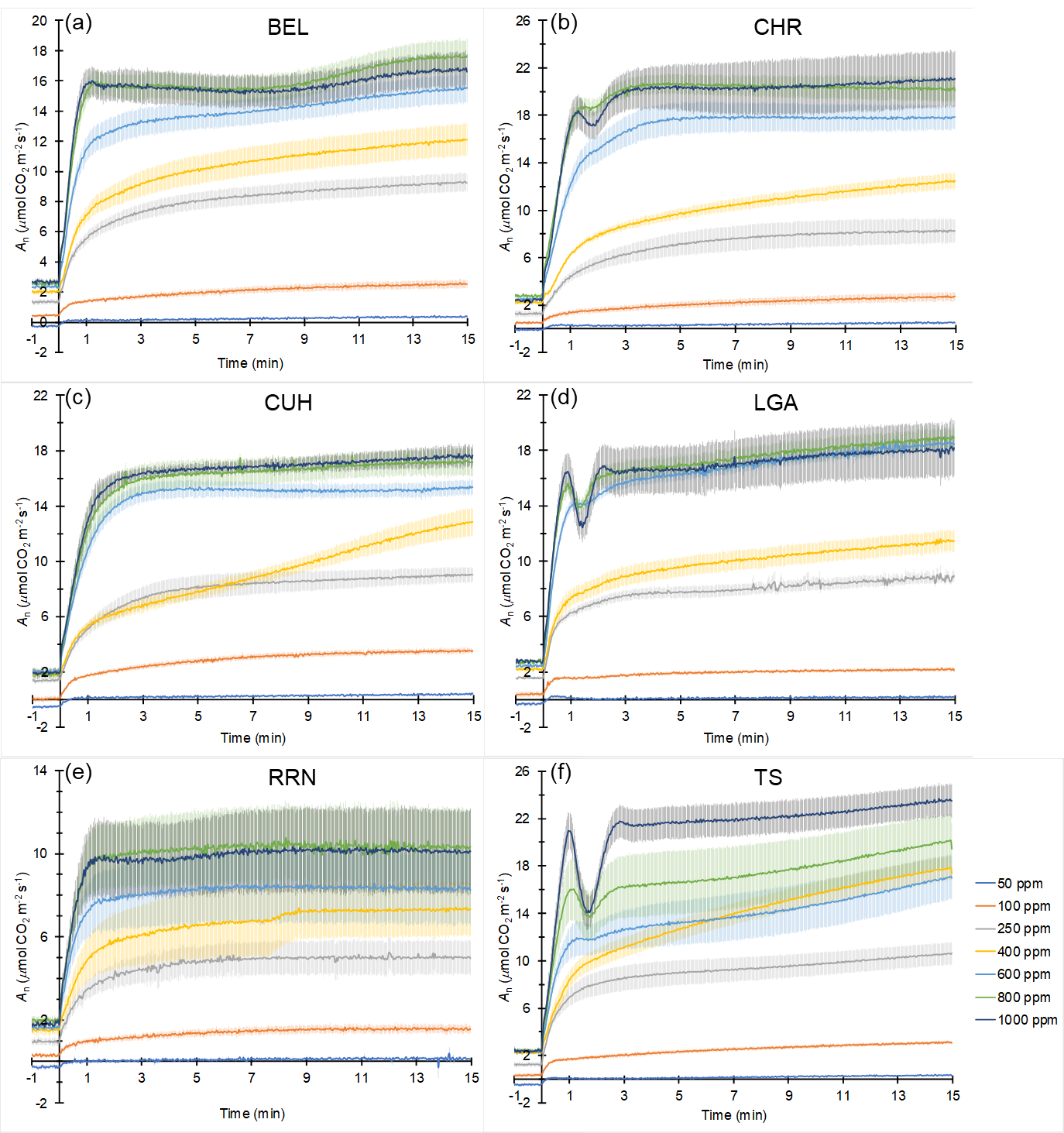
Figure S11. Photosynthetic induction as a function of different ambient [CO_2_] in basil (a), chrysanthemum (b), cucumber (c), lettuce (d), rose (e) and tomato (f). For each crop, one cultivar displaying the medium T_50_ value was chosen as an example. Each curve represents the mean of 6-7 individual plants. Error bars represent standard errors of means.

**Supplementary methods**

Method S1. Description of the FvCB model.

The FvCB model (Farquhar *et al.*, 1980) predicts net leaf photosynthetic rate (*A*_n_) as the minimum of the Rubisco carboxylation-limited rate (*A*_c_) and the electron transport-limited rate (*A*_j_):

$A_{n}=min(A_{c},A_{j})$ (Eq. S1)

$A_{c}=\frac{(C_{c}-\Gamma^{*})\times V_{cmax}}{C_{c}+K_{mC}\times(1+O/K_{mO})}-R_{d}$ (Eq. S2)

$A_{j}=\frac{(C_{c}-\Gamma^{*})\times J}{4C_{c}+8\Gamma^{*}}-R_{d}$ (Eq. S3)

where *C*_c_ is the CO_2_ concentration in the chloroplast, and was assumed to be equal to intercellular CO_2_ concentration (*C*_i_) in this study (i.e. mesophyll conductance was assumed to be infinite); *O* is partial pressure of O_2_ (21 kPa); *R*_d_ is dark respiration rate, and was calculated a priori as the intercept with the y-axis of the common intersection of *A* versus C_i_ at low and high PPFD (Laisk, 1977); Γ* is the CO_2_ compensation point in the absence of R_d_ (37.4 *μ*bar at 25 °C; (Bernacchi *et al.*, 2002)); *V*_cmax_ (*μ*mol m^-2^ s^-1^) is the maximum Rubisco carboxylation rate; *J* (*μ*mol m^-2^ s^-1^) is linear electron transport rate at high irradiance; *K*_mC_ and *K*_mO_ are the Michaelis-Menten coefficients of Rubisco for CO_2_ and O_2_, respectively. *K*_mC_, *K*_mO_ and Γ* are adjusted based on leaf temperature (with *T*_leaf_ (K) = *T*_leaf_ (°C) + 273.15) during gas exchange measurements (Bernacchi *et al.*, 2002):

$X= \exp\left( c- \frac{\Delta H_{a}}{R T_{leaf}} \right)$ (Eq. S4)

where X stands for either *K*_mC_ (*μ*mol mol^-1^), *K*_mO_ (kPa) or Γ*(*μ*mol m^-2^ s^-1^). c in Eq. S4 is the normalized scaling constant of *K*_mC_, *K*_mO_ or Γ* respectively (c *K*_mC_ = 38.28; c *K*_mO_ = 12.38 (Walker *et. al*., 2013) and c Γ* = 13.49 (Bernacchi *et al.*, 2002); $\Delta H_{a}$ (kJmol^-1^) is the energy of activation of the 3 respective parameters ($\Delta H_{a}$*K*_mC_ = 80.99 ; $\Delta H_{a}$*K*_mO_ = 23.72 and $\Delta H_{a}$Γ* = 24.46 (Bernacchi *et al.*, 2002); *R* is the universal gas constant (= 0.008314 kJ K^-1^ mol^-1^). The time responses of *V*_cmax_ and *J* during photosynthetic induction were then obtained by fitting the dynamic *A*/*C*_i_ curves generated every two seconds from the photosynthetic induction measurements to Eqs. S1-S4.

*References*

**Bernacchi CJ, Portis AR, Nakano H, von Caemmerer S, Long SP**. 2002. Temperature response of mesophyll conductance. Implications for the determination of Rubisco enzyme kinetics and for limitations to photosynthesis *in vivo*. Plant Physiology **130**, 1992–1998.

**Farquhar GD, von Caemmerer S, Berry JA**. 1980. A biochemical model of photosynthetic CO2 assimilation in leaves of C3 species. Planta **149**, 78–90.

**Laisk AK**. 1977. Kinetics of photosynthesis and photorespiration of C3 in plants. Nauka Moscow (in Russian).

**Walker B, Ariza LS, Kaines S, Badger MR, Cousins AB.** 2013. *In vivo* Rubisco kinetics and mesophyll conductance. Plant, Cell and Environment **36**, 2108-2119.

Method S2. Detailed steps to solve stomatal pore area from Eq. 9 in the main text.

$g_{s,max}=\frac{d\times SD\times a_{max}}{v\times(l+\frac{\pi}{2}\times\sqrt{\frac{a_{max}}{\pi}})}$ (Eq. 9 in the main text)

where *d* is diffusivity of water vapour in air; *v* is molar volume of air; SD is stomatal density; *a*_max_ is the maximum pore area and is approximated as *π*(*ρ*/2)^2^, where *ρ* is stomatal pore length and *l* is stomatal pore depth (assumed to be equal to guard cell width). When substituting *g*_s,max_ in Eq. 9 with *g*_s_ obtained from gas exchange measurements, *a*_max_ represents the average stomatal pore area *a* across the leaf surface.

$g_{s}=\frac{d\times SD\times\alpha}{v\times(l+\frac{\pi}{2}\times\sqrt{\frac{\alpha}{\pi}})}$

Making $b=\sqrt{\alpha}$ gives Eq. S1:

$g_{s}=\frac{d\times SD\times b^{2}}{v\times(l+\frac{\pi}{2}\times\frac{b}{\sqrt{\pi}})}$ (Eq. S1)

A transformation of Eq. S1 gives Eq. S2:

$d\times SD\times b^{2}-g_{s}\times v\times\frac{\pi}{2}\times\frac{b}{\sqrt{\pi}}-g_{s}\times v\times l=0$ (Eq. S2)

Making $x=d\times SD$ (Eq. S2.1)

$y=g_{s}\times v\times\frac{\pi}{2}\times\frac{1}{\sqrt{\pi}}=\frac{\sqrt{\pi}}{2}\times g_{s}\times v$ (Eq. S2.2)

$z=g_{s}\times v\times l$ (Eq. S2.3)

Eq. S2 can be transformed to Eq. S3:

$x\times b^{2}-y\times b-z=0$ (Eq. S3)

Solve *b* from Eq. S3 gives Eq. S4:

$b=\frac{y\pm\sqrt{y^{2}+4\times x\times z}}{2\times x}$ (Eq. S4)

Given that $b=\sqrt{\alpha}$ , only the positive solution should be taken, which gives Eq. S4.1:

$b=\frac{y+\sqrt{y^{2}+4\times x\times z}}{2\times x}$ (Eq. S4.1)

Thus *α* can be expressed as Eq. S5:

$\alpha={(\frac{y+\sqrt{y^{2}+4\times x\times z}}{2\times x})}^{2}$ (Eq. S5)

Substituting *x*, *y* and *z* respectively using Eq. S2.1-S2.3 gives the solution for stomatal pore area *α* (Eq. 10 in the main text):

$a={(\frac{\frac{\sqrt{\pi}}{2}\times g_{s}\times v+\sqrt{\frac{\pi}{4}\times{(g_{s}\times v)}^{2}+4\times SD\times d\times g_{s}\times v\times l}}{2\times SD\times d})}^{2}$ (Eq. 10 in the main text)
