# Supplementary material for: Variation of Photosynthetic Induction in Major Horticultural Crops Is Mostly Driven by Differences in Stomatal Traits
Source: Front Plant Sci. 2022 Apr 27;13:860229. doi: 10.3389/fpls.2022.860229 (PMC9094112; doi:10.3389/fpls.2022.860229)
Supplement: Supplementary Presentation 1 — Comparisons between the measured and predicted leaf net photosynthesis rates. [file Presentation_1.PPTX]

## Slide 1
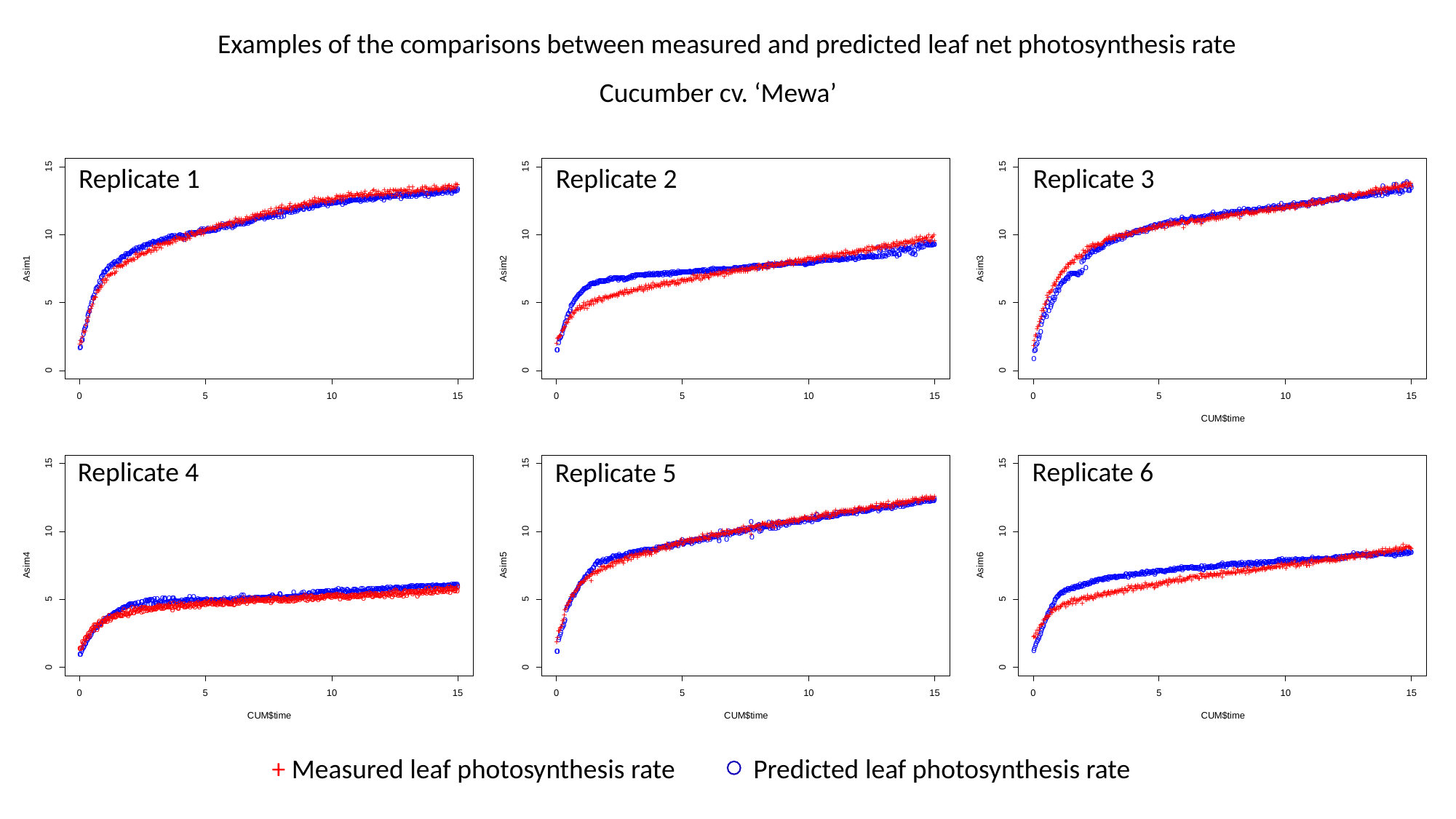

Examples of the comparisons between measured and predicted leaf net photosynthesis rate
Cucumber cv. ‘Mewa’
Replicate 1
Replicate 3
Replicate 2
Replicate 4
Replicate 6
Replicate 5
+ Measured leaf photosynthesis rate
Predicted leaf photosynthesis rate

## Slide 2
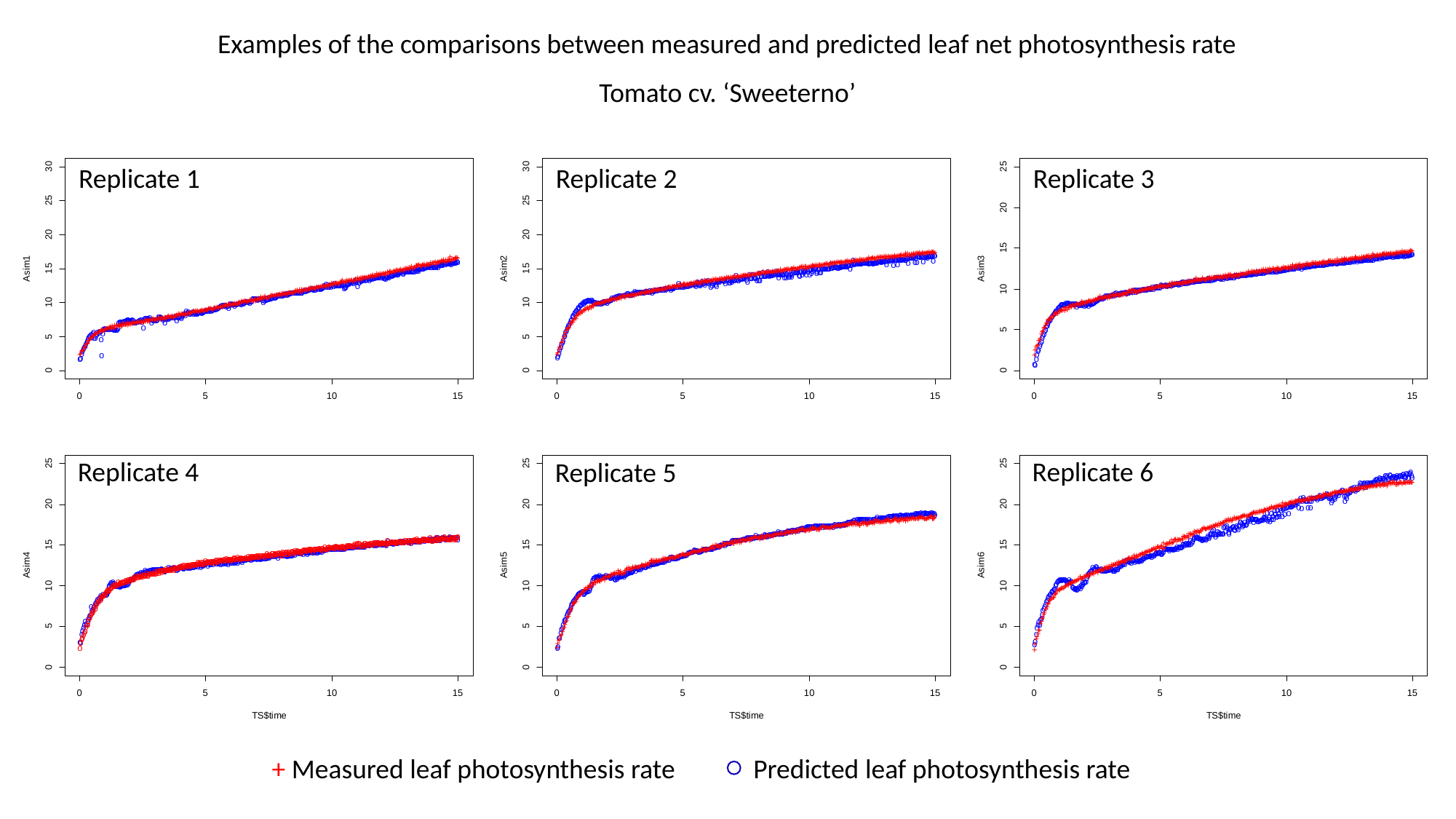

Examples of the comparisons between measured and predicted leaf net photosynthesis rate
Tomato cv. ‘Sweeterno’
Replicate 1
Replicate 3
Replicate 2
Replicate 4
Replicate 6
Replicate 5
+ Measured leaf photosynthesis rate
Predicted leaf photosynthesis rate

## Slide 3
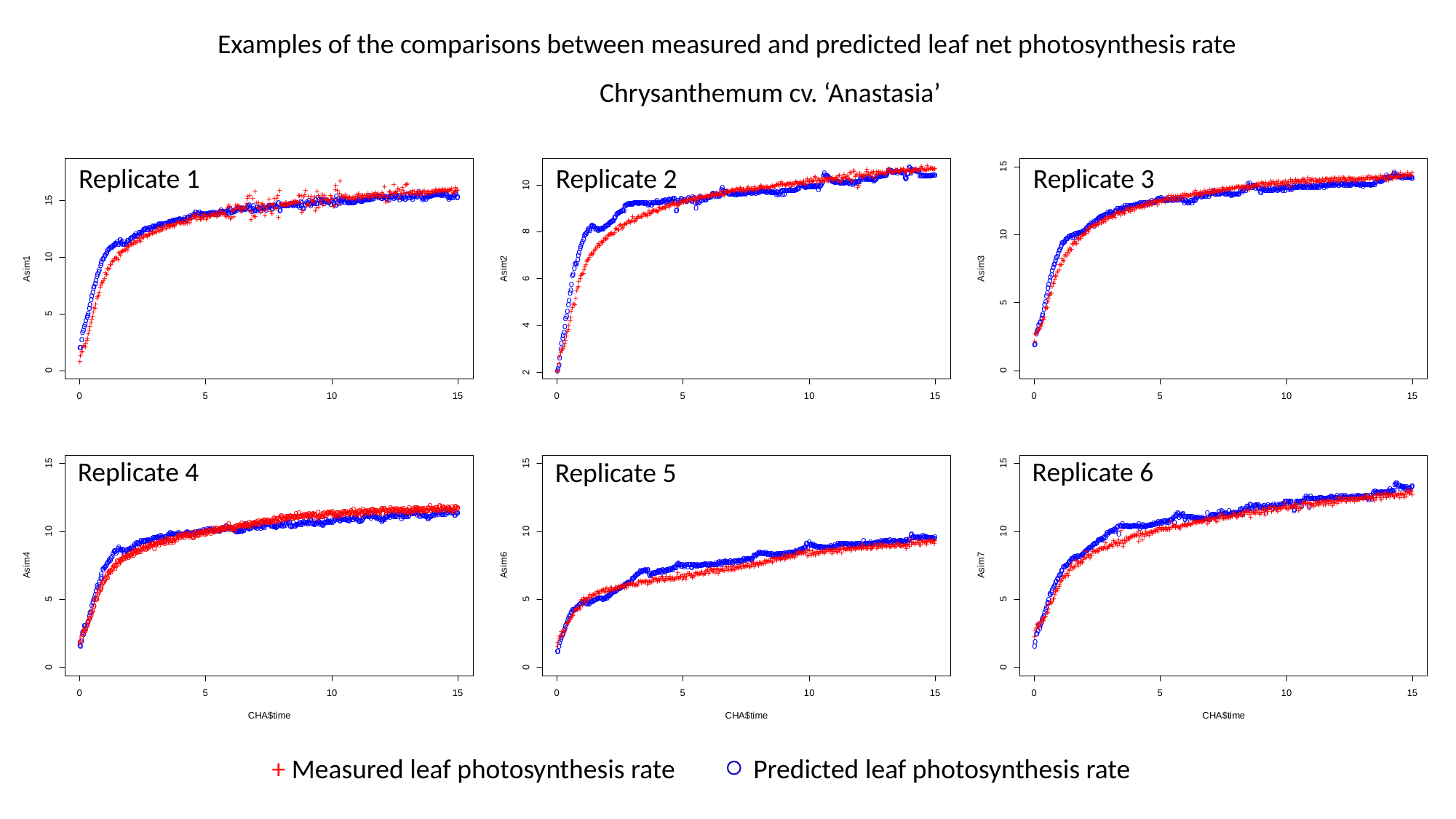

Examples of the comparisons between measured and predicted leaf net photosynthesis rate
Chrysanthemum cv. ‘Anastasia’
Replicate 1
Replicate 3
Replicate 2
Replicate 4
Replicate 6
Replicate 5
+ Measured leaf photosynthesis rate
Predicted leaf photosynthesis rate

## Slide 4
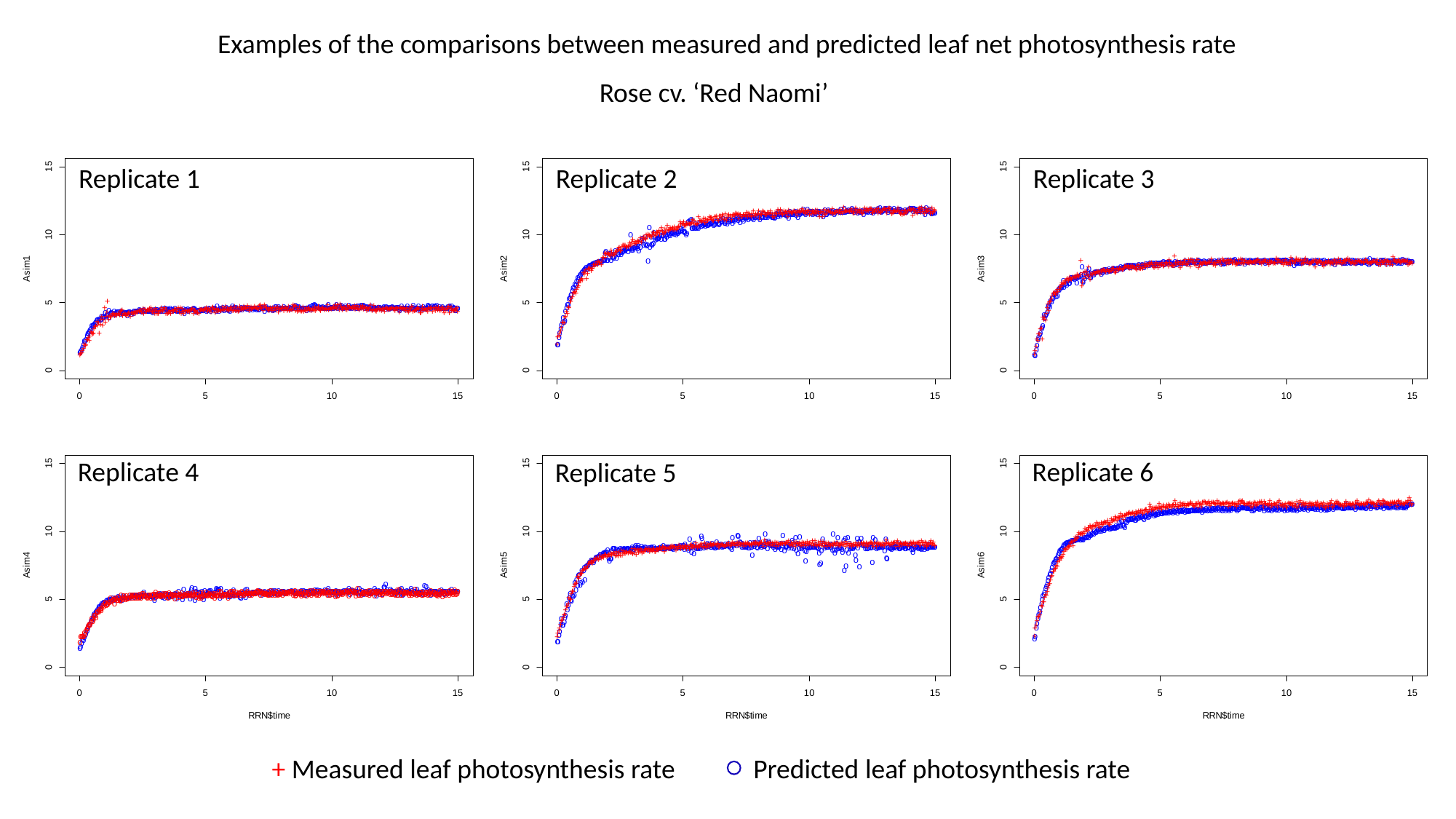

Examples of the comparisons between measured and predicted leaf net photosynthesis rate
Rose cv. ‘Red Naomi’
Replicate 1
Replicate 3
Replicate 2
Replicate 4
Replicate 6
Replicate 5
+ Measured leaf photosynthesis rate
Predicted leaf photosynthesis rate

## Slide 5
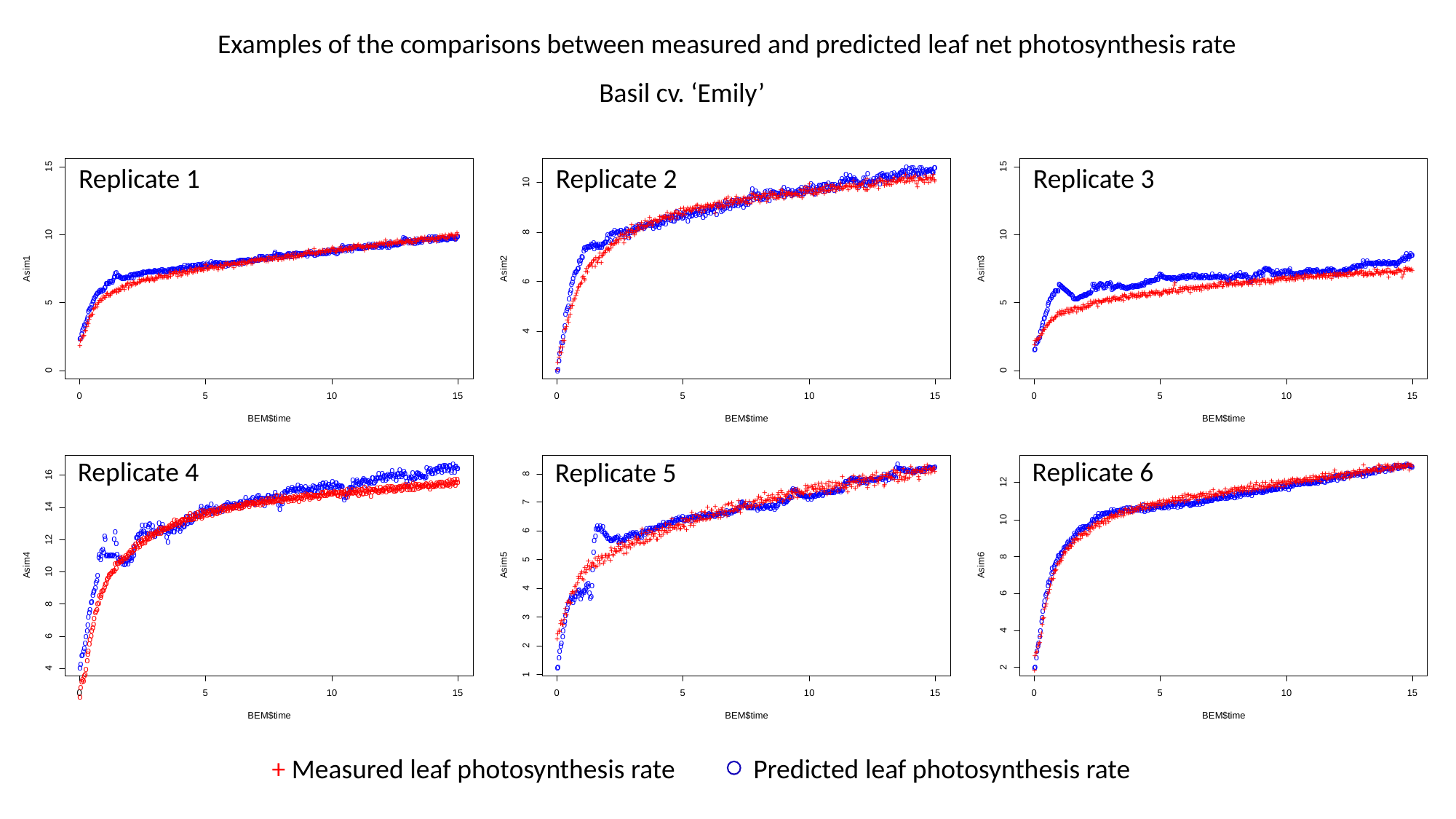

Examples of the comparisons between measured and predicted leaf net photosynthesis rate
Basil cv. ‘Emily’
Replicate 1
Replicate 3
Replicate 2
Replicate 4
Replicate 6
Replicate 5
+ Measured leaf photosynthesis rate
Predicted leaf photosynthesis rate

## Slide 6
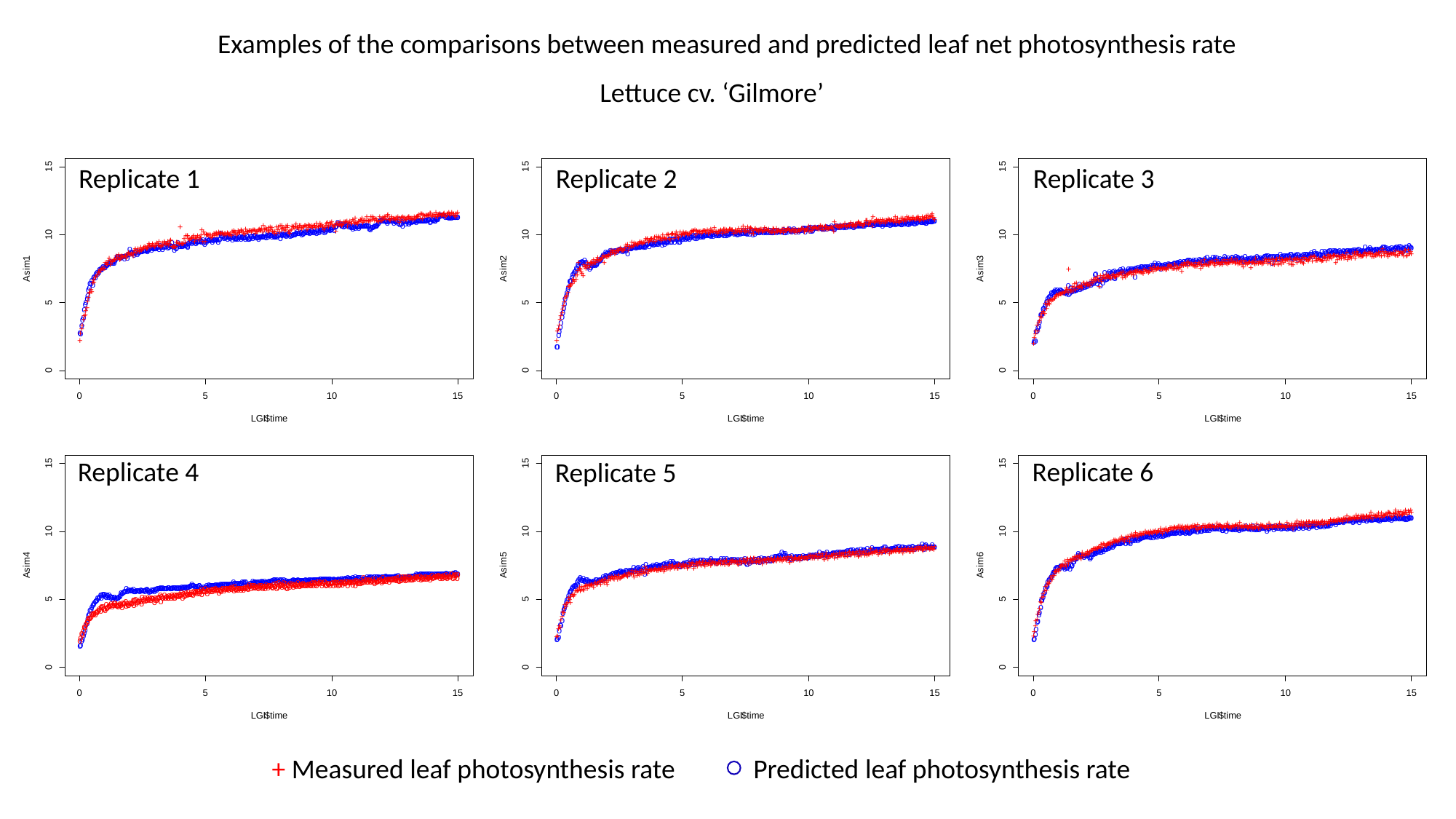

Examples of the comparisons between measured and predicted leaf net photosynthesis rate
Lettuce cv. ‘Gilmore’
Replicate 1
Replicate 3
Replicate 2
Replicate 4
Replicate 6
Replicate 5
+ Measured leaf photosynthesis rate
Predicted leaf photosynthesis rate
